# Optical mapping reveals a higher level of genomic architecture of chained fusions in cancer

**DOI:** 10.1101/gr.227975.117

**Published:** 2018-05

**Authors:** Eva K.F. Chan, Daniel L. Cameron, Desiree C. Petersen, Ruth J. Lyons, Benedetta F. Baldi, Anthony T. Papenfuss, David M. Thomas, Vanessa M. Hayes

**Affiliations:** 1 Genomics and Epigenetics Division, Garvan Institute of Medical Research, New South Wales 2010, Australia;; 2 St Vincent's Clinical School, University of New South Wales, New South Wales 2052, Australia;; 3 Bioinformatics Division, The Walter and Eliza Hall Institute of Medical Research, Victoria 3052, Australia;; 4 Department of Medical Biology, University of Melbourne, Victoria 3010, Australia;; 5 Department of Mathematics and Statistics, University of Melbourne, Victoria 3010, Australia;; 6 Sir Peter MacCallum Department of Oncology, University of Melbourne, Victoria 3010, Australia;; 7 Bioinformatics and Cancer Genomics, Peter MacCallum Cancer Centre, Victoria 3002, Australia;; 8 The Kinghorn Cancer Centre, Garvan Institute of Medical Research, New South Wales 2010, Australia;; 9 Cancer Division, Garvan Institute of Medical Research, New South Wales 2010, Australia;; 10 School of Health Systems and Public Health, University of Pretoria, Hatfield 0002, South Africa;; 11 Central Clinical School, University of Sydney, New South Wales 2006, Australia

## Abstract

Genomic rearrangements are common in cancer, with demonstrated links to disease progression and treatment response. These rearrangements can be complex, resulting in fusions of multiple chromosomal fragments and generation of derivative chromosomes. Although methods exist for detecting individual fusions, they are generally unable to reconstruct complex chained events. To overcome these limitations, we adopted a new optical mapping approach, allowing megabase-length genome maps to be reconstructed and rearranged genomes to be visualized without loss of integrity. Whole-genome mapping (Bionano Genomics) of a well-studied highly rearranged liposarcoma cell line resulted in 3338 assembled consensus genome maps, including 72 fusion maps. These fusion maps represent 112.3 Mb of highly rearranged genomic regions, illuminating the complex architecture of chained fusions, including content, order, orientation, and size. Spanning the junction of 147 chromosomal translocations, we found a total of 28 Mb of interspersed sequences that could not be aligned to the reference genome. Traversing these interspersed sequences using short-read sequencing breakpoint calls, we were able to identify and place 399 sequencing fragments within the optical mapping gaps, thus illustrating the complementary nature of optical mapping and short-read sequencing. We demonstrate that optical mapping provides a powerful new approach for capturing a higher level of complex genomic architecture, creating a scaffold for renewed interpretation of sequencing data of particular relevance to human cancer.

Structural variation (SV) is a type of genomic variation characterized by deletions, insertions, inversions, and translocations of large genomic fragments (>1 kb). Although SV is a form of natural polymorphism (Sudmant et al. 2015; The 1000 Genomes Project Consortium 2015), specific or excessive aberrations have been linked to numerous human diseases (Lupski 1998; Weischenfeldt et al. 2013). In cancer, acquired SVs have been used for molecular subclassification and shown to be predictive of cancer progression and treatment response (Hicks et al. 2006; Holland and Cleveland 2012; Baca et al. 2013; Andersson et al. 2016; Papaemmanuil et al. 2016). Beyond isolated SVs, large complex genomic rearrangements (CGRs) or chained fusions, defined as aberrant joining of multiple distant genomic regions (Malhotra et al. 2013), have been implicated in 5%–9% of all cancers (Dzamba et al. 2017). Several models of CGR have been proposed, each thought to be preferential in different cancer types. Chromothripsis, characterized by highly localized shattering and religation (fusion) of tens to hundreds of DNA fragments and an oscillating copy number profile, is, for example, present in 25% of bone cancers (Stephens et al. 2011). Chromoplexy, characterized by closed chains of chromosomal translocations, with little to no copy number alteration, is a key characteristic of prostate cancer (Baca et al. 2013). Breakage-fusion-bridge amplification, characterized by telomere shortening and cycles of fusion, amplification, and deletion, has been implicated in almost every cancer type (McClintock 1941). Despite these distinct models, it is clear that many cancers are probably driven by a combination, and likely intermediate modified forms, of these structural rearrangement processes (Zhang et al. 2013; Garsed et al. 2014).

The complexity of chromosomal abnormalities is typically inferred using various statistical and computational approaches, based on data from karyotyping, microarray-based copy number profiling, and whole-genome paired-end sequencing. A major limitation of these methods is their inability to accurately reconstruct the high levels of amplifications and complex patterns of chained fusions. Reconstruction of a rearranged genome (i.e., to “walk” a derivative chromosome) primarily requires the integration of predicted breakpoints, rearrangement signatures (orientation of sequence read alignment), and copy number profile. However, in more realistic scenarios in which derivative chromosomes are formed from multiple processes, rearrangement signatures become confounded and algorithmic assumptions breakdown, making it challenging to accurately reconstruct chromosomal rearrangements (Zhang et al. 2013). Recent advances in long-read and linked-read sequencing (Clarke et al. 2009; Roberts et al. 2013) along with accompanying bioinformatics developments (Liu et al. 2017; Spies et al. 2017) are showing promising utility in this area (Bell et al. 2017; Cretu Stancu et al. 2017; Greer et al. 2017). However, to obtain sufficient coverage for multiallelic rearrangements remains cost-prohibitive with long-read sequencing (Shi et al. 2016), and immature bioinformatics tools limit link-read sequencing utility for now (Marks et al. 2017).

In this study, we demonstrate the utility of a nonsequencing approach, namely, optical genome mapping, in capturing chained fusions. We validate the feasibility of using the Irys optical mapping system from Bionano Genomics to capture extensive CGRs in a previously reported well-differentiated liposarcoma cell line T778, commonly known as 778, derived from an elderly woman with retroperitoneal relapse (Pedeutour et al. 1999). Multicolor fluorescence in situ hybridization showed substantial translocations in this cell line, and short-read sequencing of two flow-isolated neochromosome (derivative chromosome) isoforms further confirmed extensive rearrangements with a predominance of intra-chromosomal translocations (Garsed et al. 2014). We integrate whole-genome mapping with short-read sequencing to further refine the reconstruction of complex genomic rearrangements.

## Results

### Whole-genome optical mapping and structural variant detection

**Figure 1.**
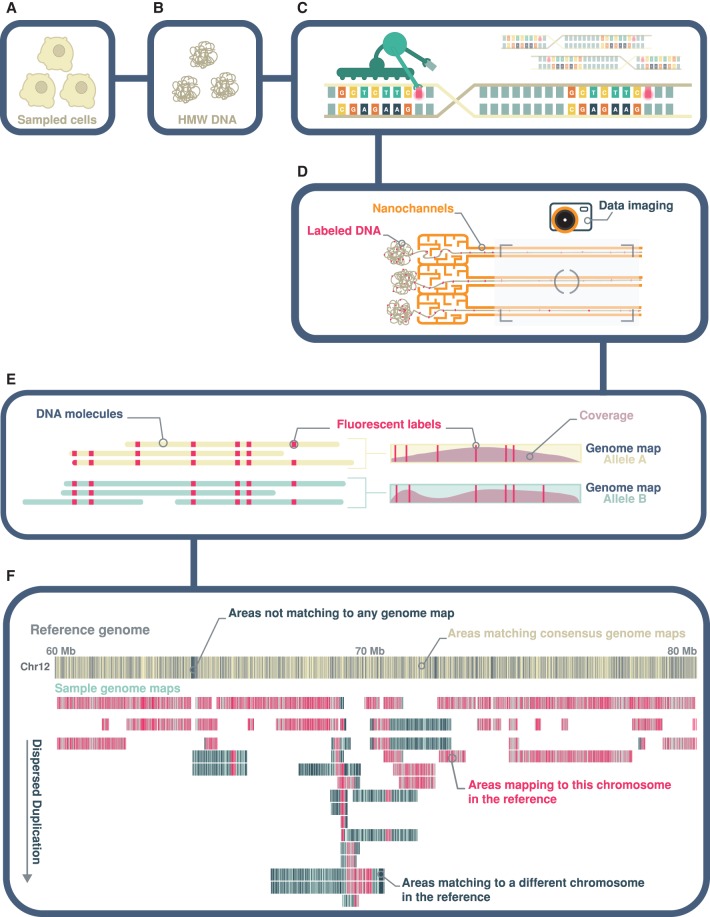
Whole-genome optical mapping. (*A*,*B*) Genome mapping begins with the extraction of intact, high molecular weight (HMW) double-stranded DNA. (*C*) An endonuclease (Nt.BspQI) that cleaves only one strand of a double-stranded DNA is used to incorporate a fluorescent dye at recognition motifs (GCTCTTCN↓) at a density of 8–11 labels per 100 kb. The DNA backbone is also stained with a second fluorescent dye, YOYO-1. (*D*) Labeled molecules are linearized, flowed into nanochannels, and imaged using the Bionano Irys instrument. (*E*) Imaged molecules are digitized and bioinformatically assembled into consensus genome maps and haplotype-phased as appropriate. (*F*) SVs relative to a reference genome are deduced based on differences in size and/or label patterns. Highly rearranged genome maps will align piecewise to multiple reference genomic regions. Conversely, heavily rearranged regions of the reference genome will show alignments from multiple genome maps (dispersed duplication). In this study, the reference genome map is an in silico digest of the human reference, GRCh38. In this figure, sample consensus genome maps are represented as teal-colored horizontal bars, overlaid with coverage density plots in mauve, whereas GRCh38 Chr 12 is shown as a gray horizontal bar. Fluorescent labels of the Nt.BspQI motif are shown as yellow and pink vertical lines overlaid on the reference and sample genome maps, respectively. Labels on the reference not aligned to any consensus genome map and labels on sample genome maps not aligned to the displayed reference chromosome(s) are shown in dark blue.

The Bionano platform is a next-generation optical mapping system for generating physical maps (genome maps) of kilobases to megabases in length (Fig. 1). This is achieved by fluorescently tagging and imaging endonuclease motif sites of single molecules in a massively parallel fashion (Hastie et al. 2013). Imaged molecules are digitized and de novo assembled into consensus genome maps, each representing the fingerprint of a large fragment of the sample genome. By comparing sample consensus genome maps against a reference genome, large SVs can be readily identified and visualized. The resolution of this technology is dependent on the frequency of the endonuclease motif in the sample genome. For a human genome, this is between 8 and 11 motifs (labels) per 100 kb using the enzyme Nt.BspQI (GCTCTTCN↓). To ensure a high level of specificity when merging and aligning molecules, de novo assembly is typically performed on a filtered set of molecules longer than 150 kb in length. Note that, unlike high-throughput sequencing, this method does not require DNA fragmentation, insert size selection, or amplification, because it is conversely reliant on intact high molecular weight DNA extraction. Although this technology has been available for several years, there had been a prevailing difficulty to accurately predict translocations and inversions. However, a recent breakthrough in bioinformatics development has made possible not only the accurate detection of inversions and translocations, but also the phasing of all detected SVs (Hastie et al. 2017).

**Figure 2.**
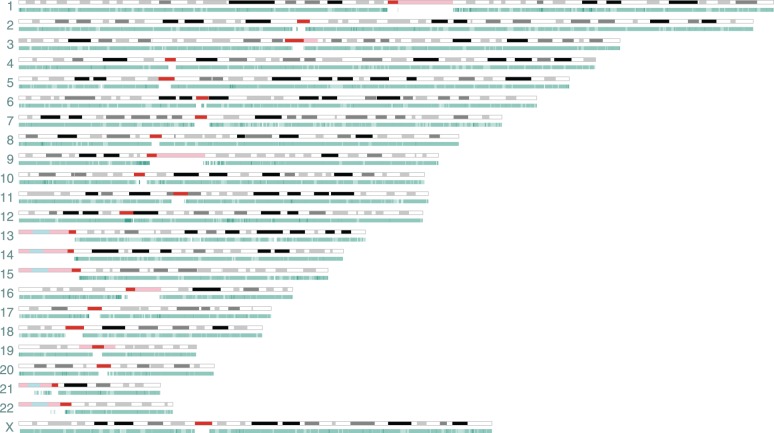
Consensus genome map overview. An overview is shown of the 3338 consensus genome maps (teal), from cell line 778, aligned to the human reference genome, GRCh38. Overlapping alignments to GRCh38 (i.e., alignment density) is reflected in the color gradient of the genome maps. The reference genome is represented by its cytogenetic G banding (UCSC Table cytoBandIdeo, last updated June 11, 2014), in which dark to light gray in this figure correspond to Giemsa stain intensity, centromeric (acen) bands are represented in red, variable heterochromatic region (gvar) in pink, and stalks (tightly constricted regions) in blue. The breadth of coverage is theoretically limited to 90% of the reference genome due to uninformative regions, including acen, gvar, and stalks.

To evaluate the ability of optical mapping for the detection of CGR, we performed whole-genome mapping on the 778 liposarcoma cell line (Pedeutour et al. 1999; Garsed et al. 2014). It is important to point out that genome mapping was performed without first isolating the neochromosomes as in the original sequencing study (Garsed et al. 2014). In summary, a total of 798,063 molecules longer than 150 kb were captured and de novo assembled into 3338 consensus genome maps (Table 1; Supplemental Data S1). From multicolor fluorescence in situ hybridization, we know most chromosomes of this cell line are tetraploid. Although the current genome mapping assembly algorithm is agnostic to ploidy greater than two, we were able to deduce an alternate haplomap (haplotype genome map) for 68% (2271/3338) of the consensus genome maps. With higher coverage and better assembly algorithm, we can expect more of the consensus maps to be further decomposed into their respective constitutive haplotypes. The average consensus genome map is 1.7 Mb in length with the longest map reaching 6.5 Mb. More than 97% of the genome maps could be aligned to the human reference GRCh38 (Schneider et al. 2017), achieving a breadth of coverage of 90%, which is a theoretical limit given the lack of reference genomic information at and around low complexity regions (Fig. 2).

**Table 1.**
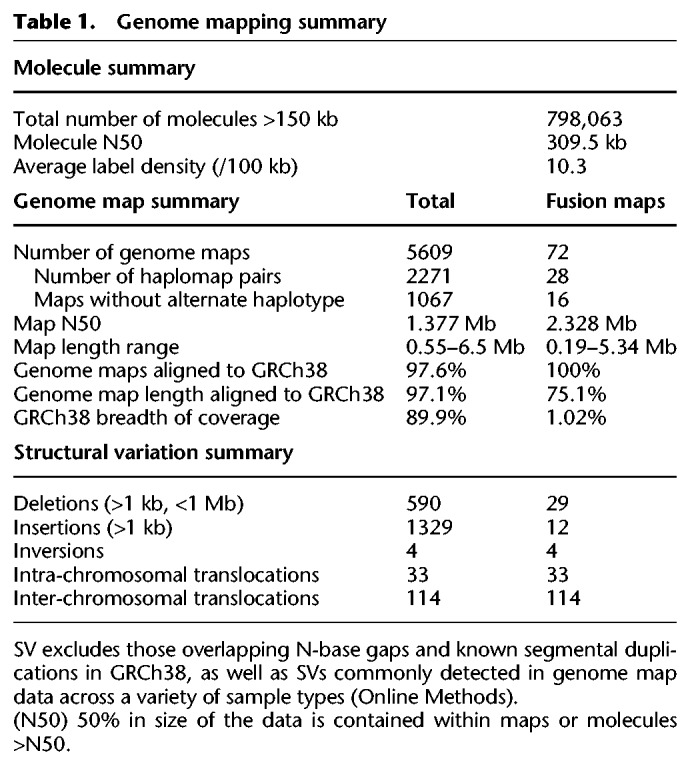
Genome mapping summary

**Figure 3.**
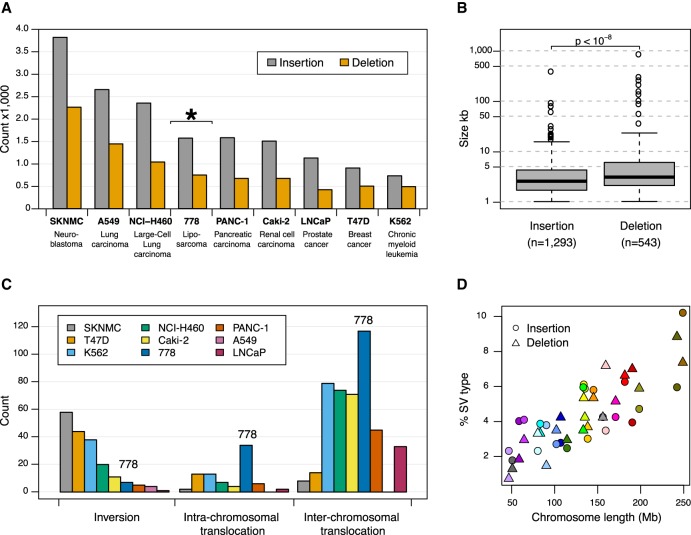
Structural variations identified using whole-genome optical mapping. (*A*,*C*) Comparison of large SVs identified in liposarcoma cell line 778 with eight other cancer cell lines reported in Dixon et al. (2017). All SVs from both studies were determined using the Bionano Irys optical mapping system. *A* shows the typical 1.5- to 2.7-fold more insertions relative to deletions with cell line 778 highlighted with an asterisk (*), whereas *C* shows cell line 778 harbors much more intra- and inter-chromosomal translocations relative to other cancer cell lines. (*B*) Box plot showing a statistically significant difference between deletion and insertion sizes, for SVs >1 kb. Thick black horizontal lines in the *middle* of the box plots correspond to median values, whereas shaded gray boxes encompass the interquartile ranges. (*D*) The proportions of large insertions and deletions found in cell line 778 are correlated with chromosome length.

A total of 2070 SVs (>1 kb) relative to GRCh38 were identified (Table 1; Supplemental Table S1). This excluded all SVs with insufficient single-molecule support and SVs overlapping N-base gaps or known segmental duplications in GRCh38. Insertions and deletions <1 kb were also excluded due to a limitation to accurately estimate inter-label distances below this size (for details, see Methods). We observed 2.3 times as many large insertions (1329) compared to deletions (590). This is similar to previous reports for other cancer cell lines using the same technology, reporting between 1.5- and 2.7-fold more insertions than deletions (Fig. 3A; Zook et al. 2016; Dixon et al. 2017) and for noncancer samples (Supplemental Fig. S1). Although insertions are more abundant, they are generally smaller in size (median: 2.6 kb; interquartile range (IQR): 1.7–4.3 kb) relative to deletions (median: 3.1 kb; IQR: 2.1–6.0 kb) (Fig. 3B). The largest insertion observed was 386 kb within Chr 1: 172,422–172,618 kb, which upon inspection is, in fact, a replacement of the 196-kb interval with a chained fusion of a 84.2-kb fragment from Chromosome 1 (188.314–188.394 Mb), a 143.6-kb fragment from Chromosome 15 (98.430–98.573 Mb), and a 354-kb fragment that does not align to GRCh38 (see below for further discussion on unaligned genome map fragments) (Supplemental Fig. S2A). The largest deletion of 850 kb at Chr 15: 90,881–91,870 kb was detected in two pairs of haplomaps, although we note genome maps spanning the deleted interval are also present (Supplemental Fig. S2B), suggesting this genomic fragment is not lost to the cancer genome. The distribution of large insertions and deletions is roughly random across the genome, with the proportion of each SV type being linearly correlated to chromosome size (Fig. 3C).

Besides large deletions and insertions, we identify 147 translocations (33 intra- and 114 inter-chromosomal) and four inversions (Fig. 3D). Compared to SVs identified in other cancer cell lines using the same Bionano Irys technology (Dixon et al. 2017), we note the extent of inversions is uniform (Fig. 3D). In contrast, we observe considerably more chromosomal translocations in 778 relative to the other cancer cell lines, which is likely due to the presence of neochromosomes in this cell line. Although 778 is highly rearranged, we acknowledge the increased number of translocations could be due to technical or analytical differences between the studies.

### Fusion maps and chained fusions

**Figure 4.**
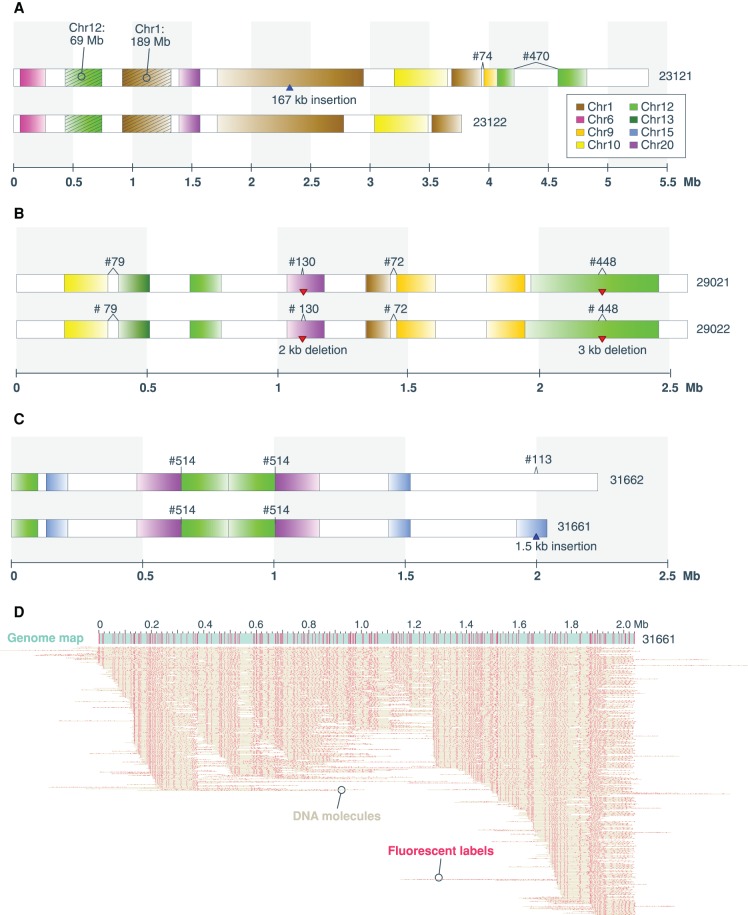
Fusion map examples. (*A*–*C*) Schematics of three fusion haplomap pairs containing complex genomic rearrangements. Genome map sizes are indicated on the horizontal axis, in megabase units. In each panel, fragments aligning to GRCh38 chromosomes are indicated by the default UCSC chromosome color scheme (color key in *A*). Uncolored (white) intervals correspond to regions not aligned to the reference. Alignment orientation to GRCh38 is indicated by color to white gradient corresponding to 5′ to 3′ alignment to the positive strand. Deletions and insertions are indicated by red downward triangles and blue upward triangles, respectively. The two most frequently represented reference fragments (Chr 1: 188,188,529–189,139,998 and Chr 12: 68,713,897–69,940,974) found in the fusion maps are shown with diagonal stripes and indicated as Chr 1: 189 Mb and Chr 12: 69 Mb. Previously identified fusions from sequencing data are numbered per Supplemental Table S1 (cf. Garsed et al. 2014) and indicated *above* the genome maps. (*D*) The molecules are aligning to, and making up, consensus genome map #31661, which contains an inverted chained fusion as shown in *C*. Here, the 2-Mb consensus genome map is represented by a teal horizontal bar following the convention in Figure 1. Individual molecules are represented as “dots on a string,” where each yellow horizontal line represents a molecule, and pink dots represent fluorescent labels. *A*–*C* are a subset of the 72 fusion maps shown in Supplemental Figure S3.

To better interrogate CGR, we focused on 72 genome maps containing all observed translocations and inversions, as well as insertions and deletions larger than 100 kb in size (Methods). This set of genome maps, termed “fusion maps,” includes 28 haplomap pairs and 16 singletons, totaling 112.3 Mb diploid length. Supplemental Figure S3 displays the schematic representation of the full set of 72 fusion maps, from which three examples are shown in Figure 4. Alignment of the fusion maps to GRCh38 revealed a total of 192 SVs, with the majority being inter-chromosomal translocations, followed by intra-chromosomal translocations, large deletions, insertions, intra-chromosomal translocations, and inversions (Table 1). Of the fusion maps, 70% have at least one inter-chromosomal translocation, and one-third have two or more (Supplemental Fig. S4). In contrast, <9% of the fusion maps contain more than one of the other types of SVs. The typical 1.5:2.5 ratio of insertions to deletions (Fig. 3A) was not observed in the fusion maps, which contain more deletions (29) than insertions (12), and deletion sizes are no longer significantly larger than insertions (Supplemental Fig. S5). This new observation may suggest different mechanistic processes driving insertions in the background and fusion genome maps. For example, it has been suggested that sizes of certain insertion and duplication types (e.g., LINE/L1 elements) differ from deletions (Nik-Zainal et al. 2016).

**Figure 5.**
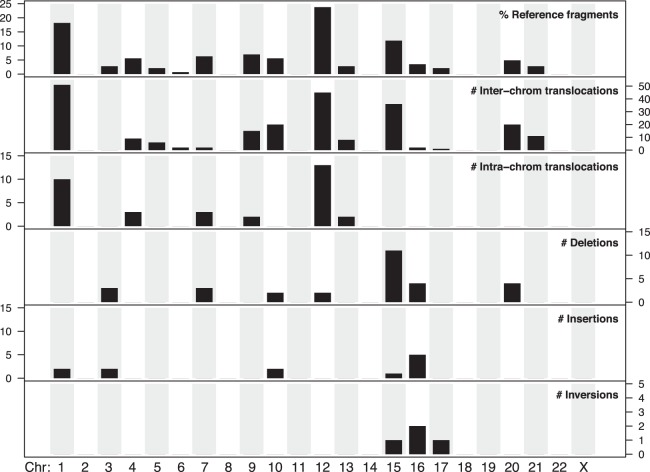
Chromosomal distribution of donor sequences and five SV types observed in 72 fusion maps. *Top* panel shows the percentage of reference donor fragments found in the fusion maps belonging to each of the 22 autosomes and Chromosome X. The next five panels show the numbers of each SV event involving the corresponding chromosomes.

Further interrogation of the fusion maps revealed extensive chained fusions (Supplemental Fig. S3). Highly duplicated and transposed reference genomic regions are notable from the pileup of genome maps aligning to these regions (Fig. 1). Chromosomes 12 and 1 are most represented, contributing 22% and 20% of all reference genome fragments found in the fusion maps, respectively (Fig. 5). Specifically, regions Chr 12: 68,713,897–69,940,974 and Chr 1: 188,188,529–189,139,998 were found in more than 10 fusion map pairs (Methods; see example in Fig. 4A). In contrast, contributions from Chromosomes 2, 8, 11, 14, 18, 19, 22, and X are notably absent. Almost all chromosomes represented in the fusion maps are impacted by translocations (Fig. 5). Of the 114 inter-chromosomal translocations, 94% involve Chromosomes 1, 12, and/or 15. Specifically, breakpoints of these translocations are largely localized to Chromosome 1: 188.6–189.2 Mb; Chr 12: 68.6–69.2 Mb; and two loci on Chr 15: 91.6–92.3 Mb and 98.2–98.7 Mb. For intra-chromosomal translocations, nearly 70% involve Chromosomes 12 and 1, with breakpoints localized to Chr 12: 68.6–69.2 Mb; Chr 12: 94.8–95.3; and Chr 1: 172.3–172.9 Mb (Supplemental Fig. S6).

**Figure 6.**
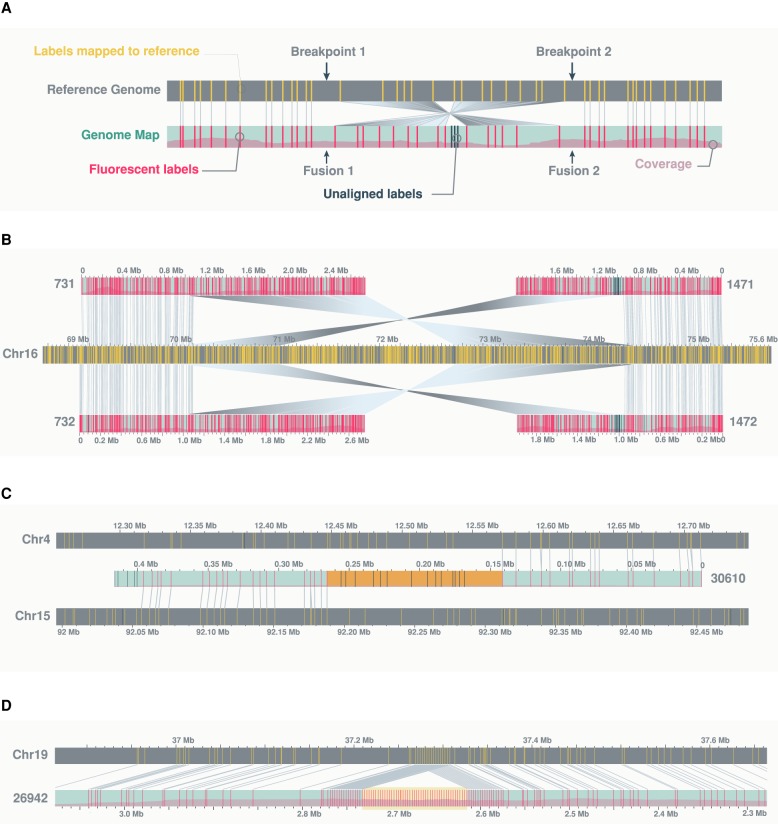
Examples of structural variants. (*A*) A schematic showing a genomic inversion as characterized by two breakpoints in the reference genome and two fusion junctions in the rearranged genome. (*B*) An example of a fully resolved 4-Mb inversion, characterized by two pairs of optical maps, each carrying one fusion junction with flanking fragments aligning in opposing directions to one side of the two reference breakpoints. (*C*) An example of a translocation between Chromosomes 4 and 15, showing “complex” label patterns at the rearrangement junction (highlighted in orange). (*D*) An example of a 159-kb insertion, showing “repeat” label patterns of the inserted fragment (highlighted in yellow). In this case, the repeat corresponds to the SST1 satellite. Additional examples highlighting complex label patterns at translocation junctions relative to repetitive label patterns of insertions can be found in Supplemental Figure S7. The schema in this figure follows the same convention as outlined in Figure 1. Matching labels between sample and reference genome maps are connected by gray lines. For clarity, any additional genome maps aligning to the reference regions of interest are hidden from view.

An inversion is typically signified by two breakpoints in the reference genome and two fusion junctions in the rearranged genome map (Fig. 6A). In one of the inversions, however, only one fusion junction was captured; thus, we can only speculate on their size. In contrast, both fusion junctions of an inversion at Chr 16: 70,195,951–74,372,427 are captured by two haplomap pairs, allowing the 4-Mb inversion to be fully defined (Fig. 6B). The remaining inversion is found on a genome map containing an inverted duplication of a series of fusion events (map #31661) (Fig. 4C), suggesting the duplication occurred after the chained fusion. Manual examination of the molecules making up the consensus genome map confirmed assembly artifact is unlikely (Fig. 4D).

Most deletions (38%) on the fusion maps involve Chromosome 15 followed by Chromosomes 16 and 7 (14% each) (Fig. 5). For large insertions, the majority involves Chromosome 16 (42%). Aside from a substantially reduced incidence of large insertions and deletions on Chromosomes 1 and 12, the distributions of these two SV types largely mirror those for translocations. Together these observations can be attributed to the rearrangement processes underlying the evolution of this cancer cell line, which included an initial catastrophic event causing the fusion of specific genomic fragments forming the cores of the derivative chromosomes (see next section).

In addition to the order and orientation of chains of donor fragments, fusion maps also captured patterns and sizes of fusion junction intervals. These intervals represent fragments that do not align to the reference genome and total ∼27.6 Mb across the 72 fusion maps. These unmapped fragments likely correspond to regions of significant rearrangements rather than repetitive elements, as their label patterns are typically “unique” (for examples, see Fig. 6C; Supplemental Fig. S7A). This is in contrast to common insertions involving repeat elements (Fig. 6D; Supplemental Fig. S7B). This theory is explored and confirmed below through the integration of short-read sequencing data.

### Fusion maps and neochromosomes

**Figure 7.**
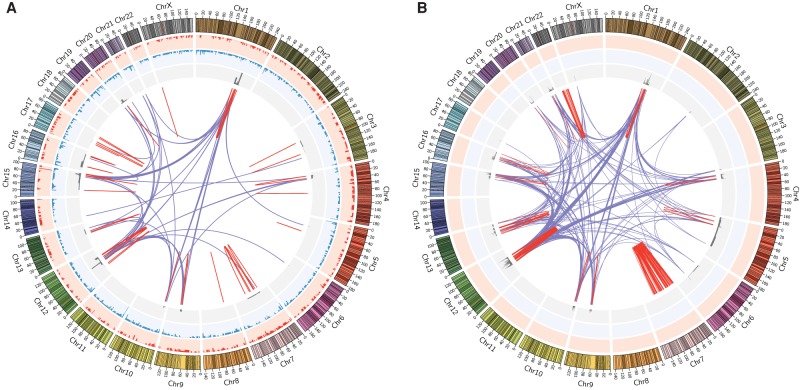
Circos plots of genomic variations in cell line 778. (*A*) Circos plot derived from whole-genome mapping of the cell line. (*B*) Circos plot derived from short-read sequencing of two neochromosome isoforms, using data from Garsed et al. (2014). For both plots, moving *inward* from the *outer* ideogram are histograms of deletions (>1 kb; red) and insertions (>1 kb; blue) in the background genome. For the neochromosome Circos plot (*B*), these two tracks are only placeholders, as the background genomes were not sequenced in the original study. The next track, in gray, shows copy number profiles of the fusion maps (*A*) and neochromosomes (*B*). Linked lines in the *middle* of the Circos plots show complex genomic rearrangements: red for intra-chromosomal and purple for inter-chromosomal translocations. Plots were generated using the Circos visualization tool (Krzywinski et al. 2009).

**Figure 8.**
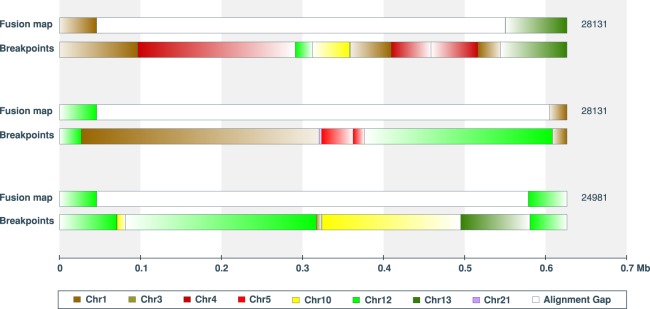
Traversal of optical mapping fusions using short-read sequencing breakpoint calls. Complex rearrangements of many small genomic fragments result in gaps in the fusion maps, because these fragments are too small to be uniquely aligned to the reference genome. Of the 86 optical mapping gaps traversed (348 kb mean gap length), the length of breakpoint traversals differed by 2024 bp on average. Shown are three examples of fusion gaps >0.5 Mb encompassing 6–9 GRIDSS breakpoints.

There are several lines of evidence suggesting that although genome mapping was performed on the entire 778 cell line, the 72 fusion maps identified largely correspond to the two neochromosome isoforms previously sequenced (Garsed et al. 2014). First, CGR and copy number profiles of the fusion maps strongly recapitulate those derived from the two neochromosome isoforms (Fig. 7; Garsed et al. 2014). A total of 21 (3.6%) of the 586 previously reported neochromosome fusions are also present in 44.4% (32/72) of the fusion maps, whereas 47 (24.5%) of the 192 CGRs identified in the 72 fusion maps were also identified from short-read sequencing of the neochromosomes (Supplemental Table S1). We attribute this low overlap to a fundamental difference in resolution between sequencing and optical mapping. Specifically, sequencing has base pair resolution allowing the detection of smaller variants, whereas mapping has resolution of about 1 kb but can detect variants that are kilo- to megabases in size. To integrate the two data sets and validate optical mapping calls, we reanalyzed the short-read sequencing data using GRIDSS, a new positional de Bruijn graph–based SV calling approach (Cameron et al. 2017) and traversed across chains following multiple breakpoints (for details, see Methods). We found support for 86 of 107 unique optical mapping fusions (Supplemental Table S2). The majority (81/86) of these confirmed fusions required the traversal of more than one GRIDSS breakpoints. On average, six sequence breakpoints were required to span an optical mapping fusion junction (Fig. 8). This supports our theory that the unmapped fusion junctions are highly rearranged sequences that have created optical mapping label patterns no longer mappable to the reference.

A second line of evidence suggesting the fusion maps may represent much of the 778 neochromosomes is in the absence of DNA fragments from Chromosomes 2, 8, 11, 14, 18, 19, 22, and X in the fusion maps (Fig. 5). This is largely consistent with the absence of both genomic fusions and contiguous genomic regions (defined as contiguous and interconnected fragments of the reference genome present in the neochromosomes) involving Chromosomes 8, 11, 14, 18, and 19 in the neochromosomes (Garsed et al. 2014). The absence of Chromosome 22 in the fusion maps is likely because this chromosome is relatively unrearranged, with the exception of a small cluster of intra-chromosomal rearrangements found in one of the neochromosome isoforms, supporting the idea that fusions involving Chromosome 22 are late events acquired after the formation of the neochromosome cores. Chromosome 2 is similarly unrearranged.

**Figure 9.**
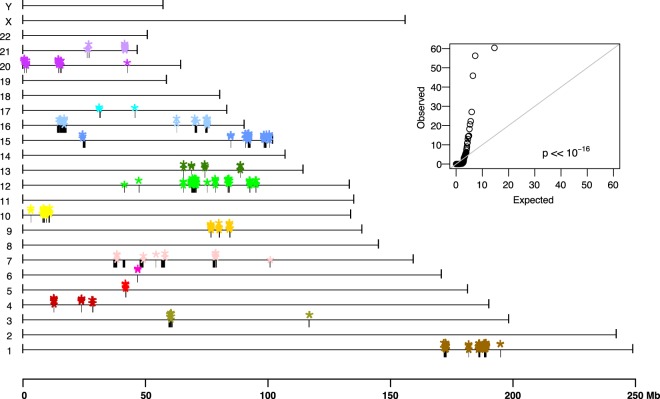
Fusion clusters. Donor fragments (black bars) and complex genomic rearrangement breakpoints (colored stars) found in the 72 fusion maps are highly localized on the reference genome map, GRCh38. Fusion breakpoints on some chromosomes are notably at the boundaries of reference donor fragments (e.g., Chromosomes 15 and 20), suggesting that acquisitions of these fragments in the rearranged genomes were late events. This is in contrast to donor fragments with “internal” CGRs (e.g., Chromosome 12), suggesting their acquisitions were early events. The *inset* is a quantile–quantile plot of the observed adjacent fusion breakpoint distances relative to the null expectation of random distribution, indicating the distribution of fusion breakpoints is statistically significantly nonrandom. Colors of plotting symbol correspond to the default UCSC chromosome color scheme.

A third line of evidence is in the dense clustering of genomic rearrangement breakpoints previously shown in the sequencing data and apparent in the fusion maps (Fig. 9). Such clustering of fusion breakpoints is characteristic of chromothripsis (Korbel and Campbell 2013). Specifically, it was theorized that the 778 neochromosomes likely resulted from an initial chromothriptic event involving Chromosome 12 leading to (1) the majority of Chromosome 12 fusions between boundaries of two islands of amplified regions being intra-chromosomal, (2) Chromosome 12 being the dominant inter-chromosomal translocation partner, and (3) the subsequent amplification of various Chromosome 12 genes by the breakage-fusion-bridge mechanism. Consistent with this, we note in the fusion maps, that (1) 39.4% of all intra-chromosomal translocations and (2) 39.5% of all inter-chromosomal translocations involve Chromosome 12, and (3) one of the two most represented reference donor fragments in the fusion maps is Chr 12: 68–70 Mb, encompassing genes *NUP107*, *YEATS4*, *MDM2*, *SLC35E3*, and *BEST3*. The active selection for the amplification of these oncogenes is a likely driver for the extensive presence of this locus in the fusion maps (Garsed et al. 2014).

Based on overlap analysis, we identified 78 reference donor fragments from the 72 fusion maps totaling 31.5 Mb (Methods). In contrast, the two 778 neochromosome isoforms are made up of 23 Mb of the reference donor genome (Garsed et al. 2014). Thus, it is plausible that some of the fusion maps identified represent the background (albeit rearranged) genome, which is consistent with observed chromosomal translocations in 778.

## Discussion

In this study, we demonstrated the utility of whole-genome mapping to reconstruct unresolved complex architectures of chained fusions. High-throughput sequencing has highlighted the importance of CGR in many human cancers. Although sequencing can identify simple SVs down to single-base variant resolution, determining the full spectrum of large CGRs remains difficult. In particular, sequencing cannot accurately reveal the physical relationships of fusion events. This is largely because CGRs are composed of multiple, often overlapping, SVs that tend to result in confounding rearrangement signatures (Zhang et al. 2013; Liu et al. 2015). This challenge is further exacerbated by inter-cell heterogeneity and aneuploidy, both hallmarks of cancer genomes. Optical mapping is designed to capture single molecules of hundreds to thousands of kilobases in size, allowing large rearrangements to be observed and phased (Dixon et al. 2017; Hastie et al. 2017; Jaratlerdsiri et al. 2017). As such, we speculate this method can provide an ideal complement to genome sequencing for resolving complex genomic architectures. We elected to test our hypothesis on a well-described, highly rearranged liposarcoma cell line 778, with known derivative chromosomes.

We performed whole-genome mapping of 778, generating 3338 assembled consensus genome maps of up to 6.5 Mb in length and recovering 72 fusion maps representing the most highly rearranged regions of the cancer genome. This allowed chained fusions to be directly observed without the need for complex algorithmic inferences reliant on intricate assumptions. This method identified 27.9-Mb genomic fragments (ranging from 40 bp to 760 kb) within fusion junctions that could not be mapped to the reference genome. Guided by optical mapping fusion events, and using a positional de Bruijin graph assembly-based SV caller and a breakpoint traversing algorithm, we were able to traverse through a sequence graph of GRIDSS-derived rearrangement breakpoints to reconstruct chains of local rearrangement events within the unmapped fusion junctions (Fig. 8). Together, these results suggest the unaligned regions represent highly fragmented genomic loci revealing the complex nature of the neochromosomes. Given the results presented here, we foresee further bioinformatics work could substantially reduce the extent of unaligned optical maps in CGRs by mapping to a reference augmented by transitive calls from short-read sequencing.

We previously proposed a process of liposarcoma neochromosome formation, initiated by chromothripsis of Chromosome 12 leading to the formation of a circular chromosome, followed by a series of breakage-fusion-bridge cycles and punctuated chromothriptic events, and ultimately terminating in a stabilized linear form (Garsed et al. 2014). The nonrandom distribution of translocations observed in this study concurs with the assumption of a single catastrophic chromothriptic-like event, occurring early in the evolution of the 778 cancer cell line. At the same time, the involvement of almost all chromosomes implies additional, plausibly stepwise, rearrangement processes along with post hoc selection were also at play following the initial catastrophic genomic event. Combining optical mapping with previous and new information from short-read sequencing and karyotyping, we gained further insight into the formation and genomic architecture of this highly rearranged cancer cell line.

Complex genomic rearrangements have a major role in cancer development, emerging as a major indicator of cancer subtype, progression, and treatment response (Hicks et al. 2006; Holland and Cleveland 2012; Baca et al. 2013; Andersson et al. 2016; Papaemmanuil et al. 2016). Comprehensively detecting CGR in cancer remains challenging. No single approach can completely identify all SV, as each approach has strengths and weaknesses. Although continued developments in long-read and linked-read sequencing are showing promise (Bell et al. 2017; Cretu Stancu et al. 2017; Greer et al. 2017; Marks et al. 2017), integrative methods combining different data types will remain imperative for the thorough elucidation of CGR (Mostovoy et al. 2016; Dixon et al. 2017).

## Methods

### Cell culture

Approximately 10 million cells of the lineage 778 were provided by A. Cipponi (Garvan Institute). This cell line was derived from a retroperitoneal relapse of a well-differentiated liposarcoma from a 68-year-old woman in 1993 (Pedeutour et al. 1999).

### Optical mapping

High molecular weight DNA was isolated using IrysPrep Plug Lysis Long DNA Isolation Protocol (Bionano Genomics). In brief, cells were trypsinized, washed in FBS/PBS, counted, rewashed in PBS, and embedded in agarose plugs using components from the Bio-Rad Plug Lysis Kit. The plugs were subjected to Proteinase K digestion (2 × 2 h at RT). After a series of washes in buffer from the Bio-Rad kit, followed by washes in TE (Tris-EDTA), the plugs were melted and treated with GELase enzyme (Epicenter). The high molecular weight DNA was released and subjected to drop dialysis. The DNA was left to equilibrate for 4 d, then quantified using the Qubit Broad Range dsDNA Assay Kit (Thermo Fisher Scientific).

Using the IrysPrep NLRS assay (Bionano Genomics), 200–300 ng/µL of high molecular weight DNA underwent single-strand nicking with 10 units of Nt.BspQI nickase (New England BioLabs). Nicked sites were repaired with fluorophore-labeled nucleotide to restore strand integrity. The backbone of fluorescently labeled double-stranded DNA was stained with the intercalation dye YOYO-1. Labeled molecules were directly loaded onto IrysChip, without further fragmentation or amplification, and imaged using the Irys instrument. Multiple cycles were performed to reach average raw genome depth of coverage of 70×.

### Consensus genome map assembly

All Bionano software used and described in this paper is freely available from the Bionano Genomics support website (https:// bionanogenomics.com/support).

Imaged molecules, in the form of TIFF files, were digitized using the software AutoDetect v2.1.4 (Bionano Genomics) into BNX files (format v1.2), which are flat text files containing, for each molecule, the length of the DNA (predicted from the DNA backbone stain), relative positions of label sites (from the NLRS step), and quality scores (based on the relative fluorescence of the dye and background). Raw DNA molecules from the BNX files were filtered based on minimum molecule length of 150 kb, minimum of nine label sites per molecule, and a signal-to-noise ratio between label and background fluorescence greater 2.75. Filtered BNX files from across multiple Irys runs were merged and provided as part of Supplemental Data S1 (all.bnx).

De novo assembly of single molecules into consensus genome maps were performed with the Bionano Solve v3.0 software (Lam et al. 2012; Hastie et al. 2013, 2017). This software includes a custom aligner (RefAligner v6159) specific for the Bionano optical mapping data type. In brief, genome map assembly is based on an overlap–layout–consensus assembly approach, beginning with a pairwise alignment of all DNA molecules (alignment *P*-value <10^−10^) to create a layout-overlap graph, forming the initial consensus genome maps that are iteratively (five times) refined (refinement *P*-value <10^−11^), extended (extension *P*-value <10^−11^), and merged (merge *P*-value <10^−15^) through realignments of DNA molecules to each successive map set. The full set of parameters to Bionano Solve is included as Supplemental Data S1 (exp_ optArguments.xml).

One of several key differences between the current software (v3.0) and previous versions (IrysSolve/IrysView) is that Bionano Solve has an added haplotype-aware component, whereby at the extension stage of the assembly, coordinated misalignments from a cluster of molecules will signal the genome map to be split for independent assembly. Molecules from different alleles are “peeled off,” allowing the capacity to handle more than two alleles. It is particularly important for assembly of segmental duplication regions, where large stretches of sequence appear more than once in the genome, as in the case in genomes with neochromosomes.

### Structural variation detection

SVs were identified relative to the human reference genome, GRCh38 (Schneider et al. 2017), whose genome maps were bioinformatically deduced based on predicted Nt.BspQI motif sites. SV detection was performed using the Bionano custom SV caller (Bionano Genomics). Details of the underlying algorithm are described in the software's accompanying documentation (Document Number 30110B: *Bionano Solve Theory of Operation: Structural Variant Calling*; https://bionanogenomics.com/wp-content/uploads/2017/03/30110-Rev-B-Bionano-Solve-Theory-of- Operation-Structural-Variant-Calling.pdf). In brief, consensus genome maps were aligned to GRCh38 using a Multiple Local Alignment algorithm, and noncontiguous alignments are identified as corresponding to potential SVs. A poorly aligned or unaligned region flanked by two well-aligned regions is identified as a deletion or insertion. Two adjacent regions of a consensus genome map that align well to distinct regions of GRCh38 are classified as translocations: If the two distinct regions are >5 Mb apart on the same chromosome, the event is an intra-chromosomal translocation, and if the two regions align to different chromosomes, the event is an inter-chromosomal translocation. The full set of parameters to the SV calling step is included as Supplemental Data S1 (exp_optArguments.xml).

Four filtering steps were performed on the resulting SVs. First, SVs with insufficient single-molecule support were excluded. This was determined using the Variant Annotation Pipeline (Bionano Genomics), a new module of Bionano Solve v3.1 (details are described in the accompanying documentation 30190A: https://bionanogenomics.com/wp-content/uploads/2017/ 08/30190-Rev-A-Bionano-Solve-Theory-of-Operation-Variant-Annotation-Pipeline.pdf). This filtering step is important in catching spurious SVs resulting from consensus genome map misassemblies. In brief, a SV is confirmed if at least 10 molecules align plus or minus two labels across each variant breakpoint on the genome map. Second, SVs spanning known N-based gaps or segmental duplications (per the UCSC *gap* [last updated December 24, 2013] and *genomicSuperDups* [last updated October 14, 2014] tables) in GRCh38 were excluded. For this, we consider the full reference interval of insertions and deletions, and 10 kb plus or minus the two breakpoints for inversions and translocations. Exclusion criteria are at least one base of these SV/breakpoint intervals overlapping with N-base gaps and 20% of the SV/breakpoint intervals overlapping with SegDups. Third, insertions and deletions <1 kb in size were excluded based on recommendation from Bionano Genomics on the fact that both sensitivity and positive predictive value rapidly decline for the detection of these two SV types <1 kb (Hastie et al. 2017; Bionano Genomics Document 30110B). Here, the size of a SV is taken as the absolute difference between the breakpoint intervals on the sample and reference genome maps. Finally, translocations spanning “common translocation breakpoints” were excluded. This list of common breakpoints was provided by Bionano derived from roughly 150 control human samples and includes those observed in at least two genome maps derived from the BspQI enzyme as well as in at least two genome maps derived from the BssSI enzyme. These translocations are flagged as “_common” by the Bionano Solve v3.0 software. It is worth noting that in our data set, all “_common” translocations were excluded by the second filtering step to exclude SVs overlapping N-gaps and SegDups.

### Fusion genome maps

Fusion maps were defined as consensus genome maps with at least one CGR, including all translocations and inversions as well as insertions and deletions >100 kb apart (i.e., inter-breakpoint distance between sample and GRCh38 genome map is >100 kb). Genome maps without CGR, but whose haplomap partner harbor at least one CGR, were also included.

Alignments of fusion maps to GRCh38 identified reference genomic regions contributing to the composition of the 72 genome maps. To find the most represented reference fragments, all GRCh38 loci present in the fusion maps were deduced (excluding those overlapping N-gaps by any base and or known SegDups by at least 50%) and merged, resulting in 78 reference donor fragments. The numbers of fusion maps containing each of these fragments were then counted, excluding duplicate counting from haplomap pairs. Two reference regions, Chr 1: 188,188,529–189,139,998 and Chr 12: 68,713,897–69,940,974, were found in 11 pairs of fusion haplomaps. The sum of fusion map alignments across the whole genome was used as the copy number profile for the 72 fusion maps in the Circos plot (Fig. 7).

Mosaic alignments of optical maps to distinct and distant reference genomic regions are a signature of complex genomic rearrangements. A genomic fusion is characterized by a pair of neighboring alignments, on an optical map, to distinct regions of the reference genome, with breakpoints corresponding to the closest pair of aligned labels. Often, a fusion junction contains a stretch of DNA (label patterns) that does not align anywhere on the reference genome. These unaligned regions likely correspond to significantly rearranged reference fragments (potentially with additional fusions) that no longer bear a semblance to the original reference (examples in Fig. 6C; Supplemental Fig. S7).

Genomic distributions of translocations and large insertions and deletions on the 72 fusion maps were calculated as the total number of each SV type per 1-Mb windows (Supplemental Fig. S6).

### Use of neochromosome deep sequencing data

Two Supplemental data files from Garsed et al. (2014) were used in this study: Table S2 (worksheet “778 (DR)”) containing a list of genomic fusions, and Table S3 (worksheet “778_CN”) containing the copy number profiles of the two neochromosome isoforms of cell line 778. Because the original study was performed with GRCh37, genomic coordinates in these data were remapped to GRCh38, using the NCBI Genome Remapping Service (https://www.ncbi.nlm.nih.gov/genome/tools/remap). Remappings to nonassembled chromosomes (chromosomes designated with suffixes _random and _alt) and unplaced sequences (ChrU) were excluded. This resulted in the unsuccessful remapping of (1) two fusions, because one of their breakpoint pairs maps ubiquitously to three different assembled chromosomes; (2) one fusion, because one of its breakpoints cannot be remapped to GRCh38, and (3) one CGR because it cannot be remapped to GRCh38.

The copy number profile and genomic fusions in the Circos plot of Figure 7 was generated based on the remappings of these two tables. Circos plots were generated using the Circos visualization tool (Krzywinski et al. 2009).

A fusion on a fusion map is considered as concordant with a fusion on the neochromosomes (per Table S2: 778 (DR) of Garsed et al. 2014) if both breakpoints of the genome map fusion are within 100 kb of the neochromosome fusion breakpoint pair. Results of the comparison are reported in Supplemental Table S1.

### Traversing fusion junctions using GRIDSS breakpoint calls

Appreciating the different information content between sequencing and optical mapping, we sought to reanalyze the short-read sequencing data using a new approach, GRIDSS, specifically designed to identify CGRs (Cameron et al. 2017). To evaluate the optical mapping fusions, we pulled out a set of 107 unique breakpoints from the fusion maps (Supplemental Table S2). This was derived from 180 total breakpoints (from the optical map alignment file EXP_REFINEFINAL1.xmap) (Supplemental Data S1; Supplemental Table S1), which were de-duplicated based on the definition that two fusion events are duplicates if both pairs of breakpoints on the reference genome are within 10 kb of each other, and the size difference in their fusion junction intervals are also within 10 kb. This set of breakpoints was compared against a breakpoint call set derived from the Illumina short-read sequencing data (Supplemental Table S2). Breakpoints were identified in Illumina sequencing data by running GRIDSS version 1.3.4 using default parameters (Cameron et al. 2017) on the aligned BAM files used by Garsed et al. (2014). As the BAMs were aligned to GRCh37, liftOver of optical mapping coordinates from GRCh38 to GRCh37 was performed. GRIDSS breakpoints with quality score above 500 were considered. Illumina calls were considered to support the optical mapping fusion breakpoint if a path through a sequence graph generated from the Illumina breakpoint calls could be found, such that the start and end points were within 100 kb of the optical mapping calls, and the difference in sequence lengths between the optical mapping and the breakpoint traversal was <100 kb. When multiple candidate paths could be found, the path whose sequence length most closely matched the optical mapping was chosen. In all, 372 unique GRIDSS breakpoints were traversed to support a total of 86 optical mapping fusions, with up to 11 traversals identified.

### Statistical analyses

The Wilcoxon rank-sum test with continuity correction was used to assess the null hypothesis that insertion and deletion sizes have the same distributions (Fig. 3B). The null was significantly rejected for both all-genome maps (*P* < 10^−8^) and not significant for the 72 fusion maps (Supplemental Fig. S5).

The two-sample Kolmogorov–Smirnov test was used to evaluate whether neighboring fusion breakpoint distances on the reference genome deviated from the null hypothesis of random distribution (Fig. 9; Korbel and Campbell 2013).

## Data access

Input molecule data along with parameter and output files from the Bionano Solve are available as Supplemental Material to this paper. Supplemental Data File S1 is publicly available via the doi: http://dx.doi.org/10.6070/H41R6P37.

## Supplementary Material

Supplemental Material
