# Supplementary material for: Optical mapping reveals a higher level of genomic architecture of chained fusions in cancer
Source: Genome Res. 2018 May;28(5):726–38. doi: 10.1101/gr.227975.117 (PMC5932612; doi:10.1101/gr.227975.117)
Supplement: Supplemental Material [file supp_28_5_726__index.html]

Optical mapping reveals a higher level of genomic architecture of chained fusions in cancer — Supplemental Material 

# Optical mapping reveals a higher level of genomic architecture of chained fusions in cancer

## Supplemental Material

- Supplemental\_Fig\_S5.pdf
- Supplemental\_Fig\_S3.pdf
- Supplemental\_Fig\_S7.ai
- Supplemental\_Fig\_S1.pdf
- Supplemental\_Fig\_S2.ai
- Supplemental\_Fig\_S6.ai
- Supplemental\_Table\_S1.xlsx
- Supplemental\_Table\_S2.xlsx
- Supplemental\_Fig\_S4.pdf
- Supplemental\_Material.pdf
