# Supplementary figures and images for: Optical mapping reveals a higher level of genomic architecture of chained fusions in cancer
Source: Genome Res. 2018 May;28(5):726–38. doi: 10.1101/gr.227975.117 (PMC5932612; doi:10.1101/gr.227975.117)

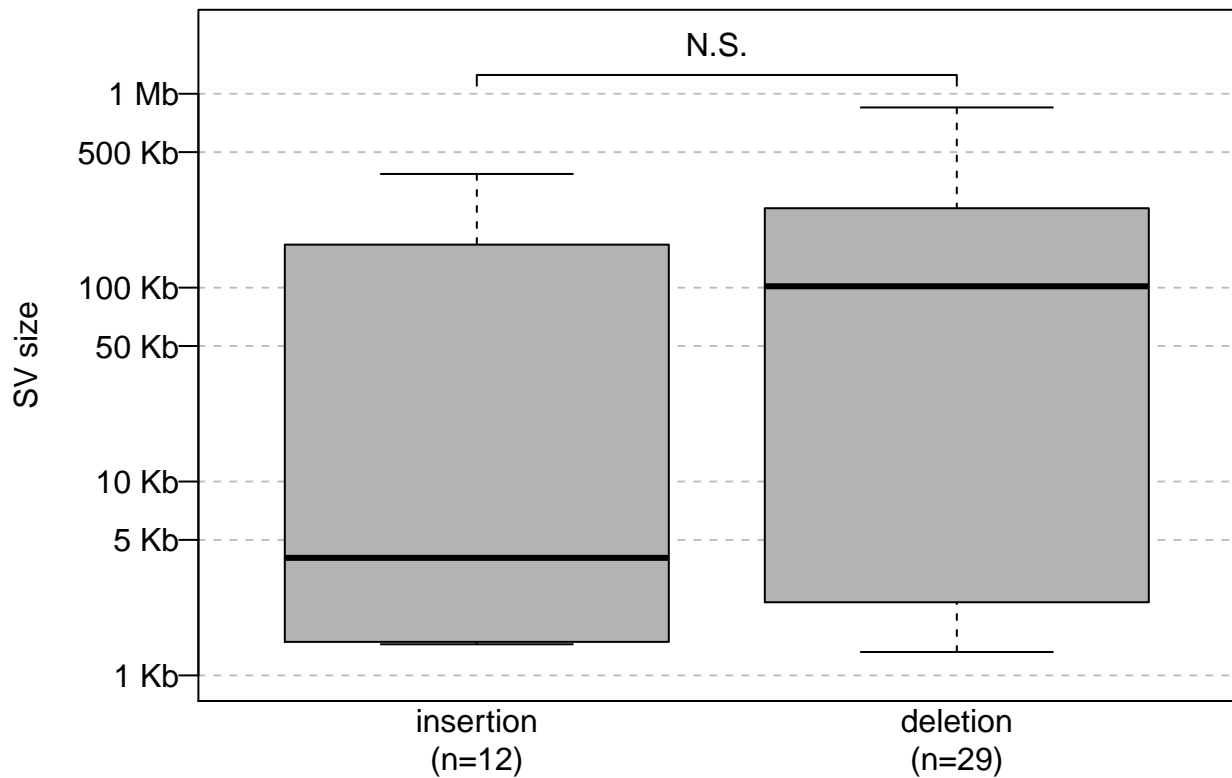

Supplement: Supplemental Material [file supp_gr.227975.117_Supplemental_Fig_S5.pdf]

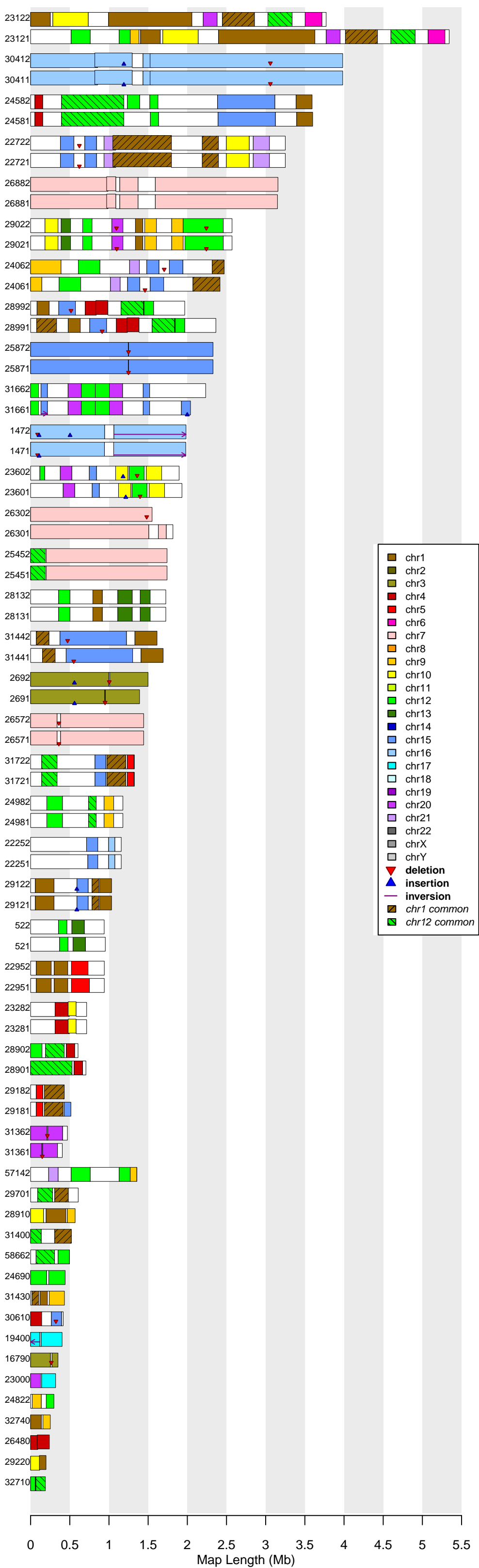

Supplement: Supplemental Material [file supp_gr.227975.117_Supplemental_Fig_S3.pdf]

**A**

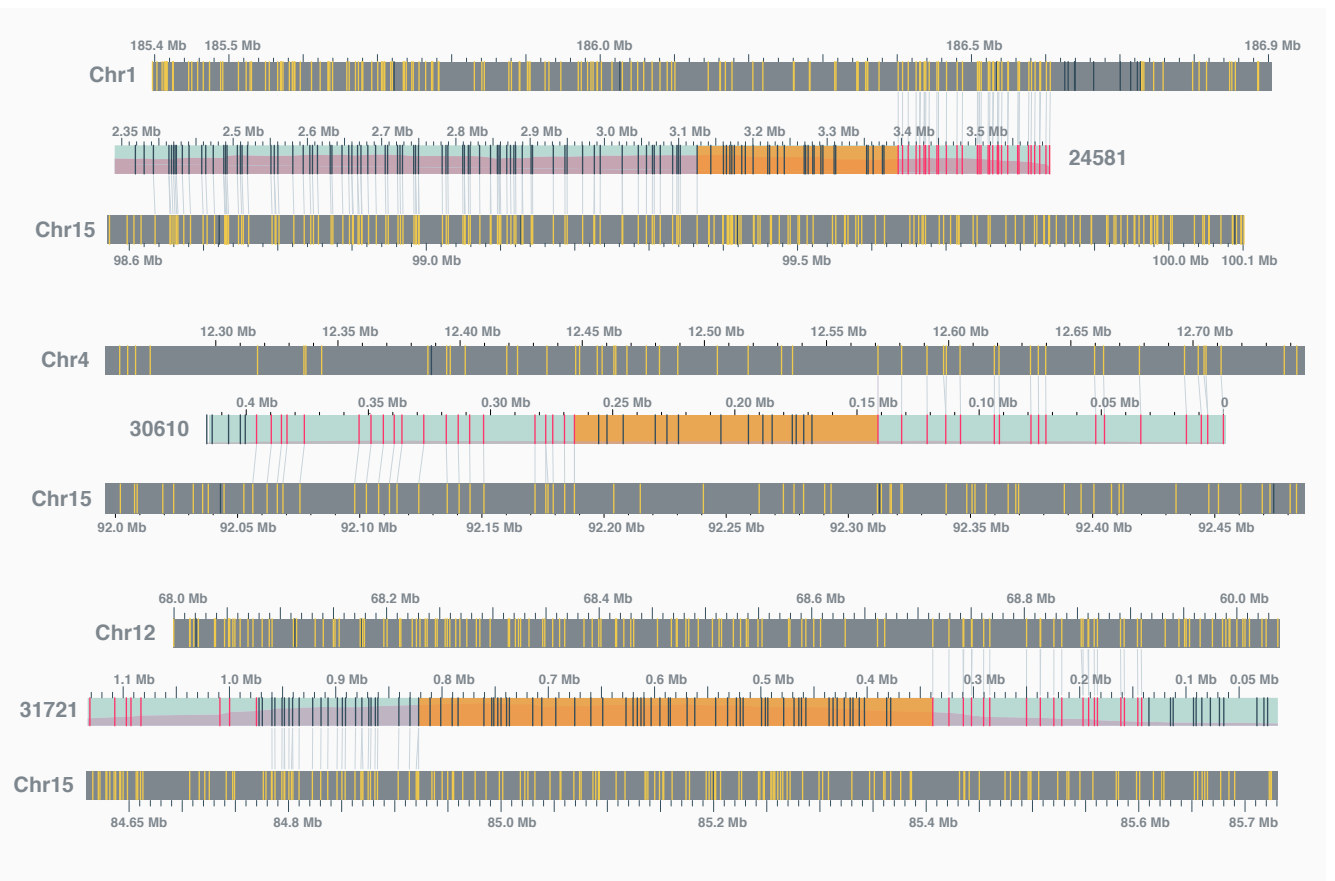

**B**

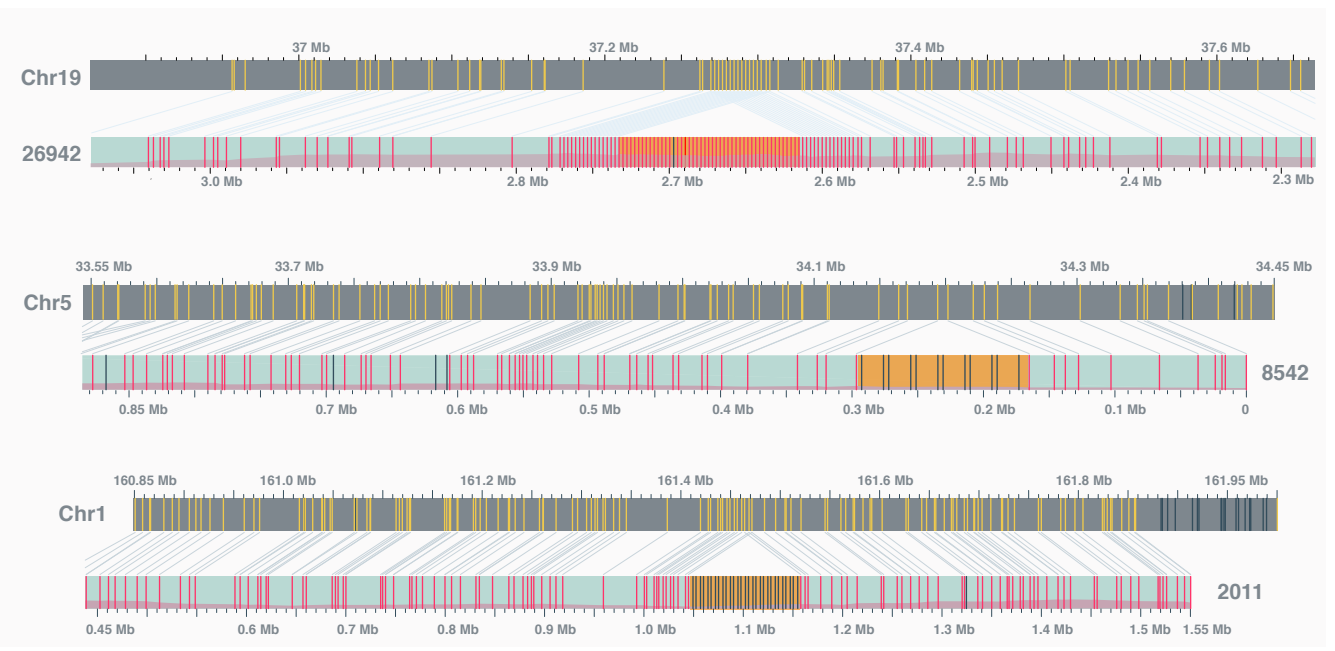

Supplement: Supplemental Material [file supp_gr.227975.117_Supplemental_Fig_S7.ps]

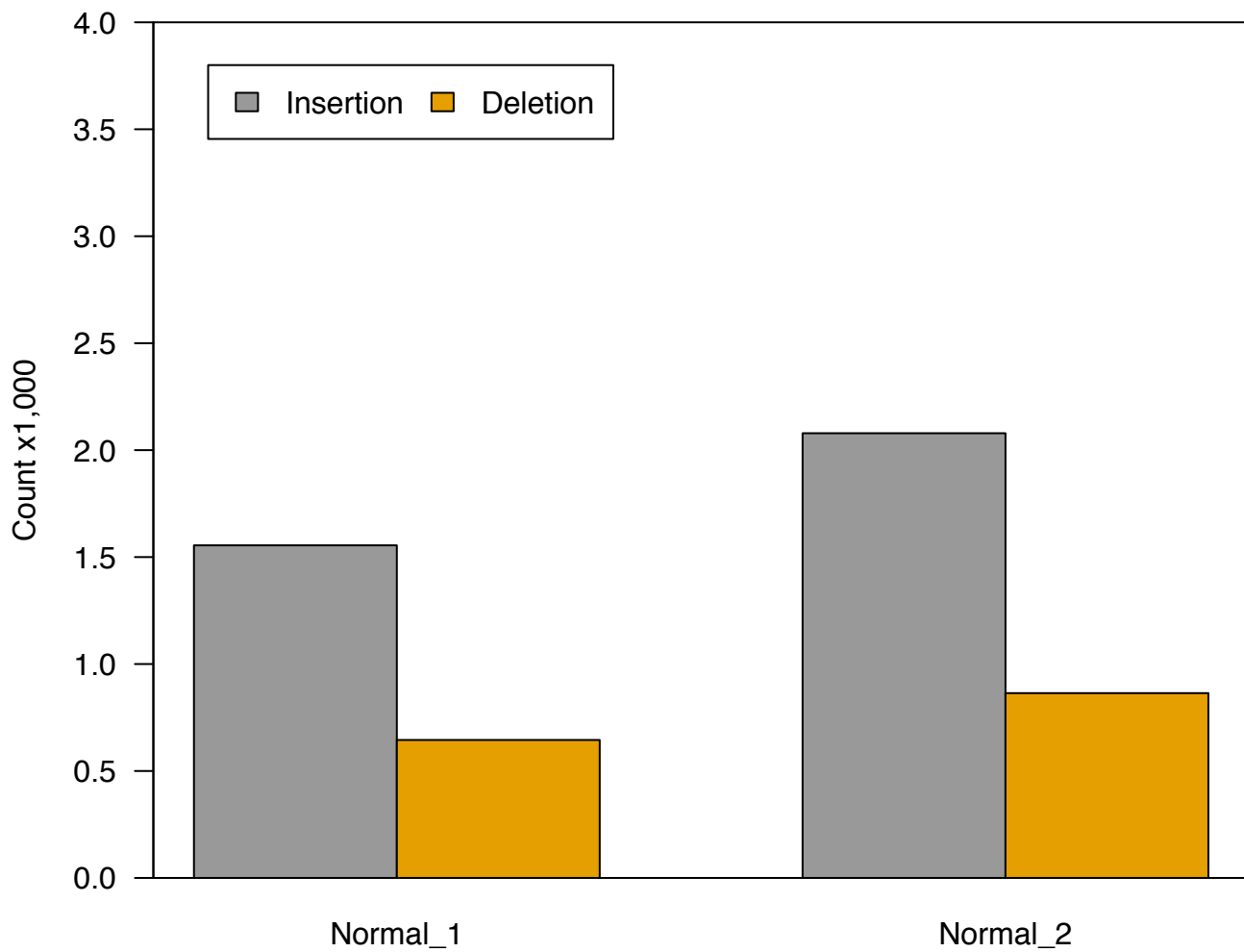

Supplement: Supplemental Material [file supp_gr.227975.117_Supplemental_Fig_S1.pdf]

A

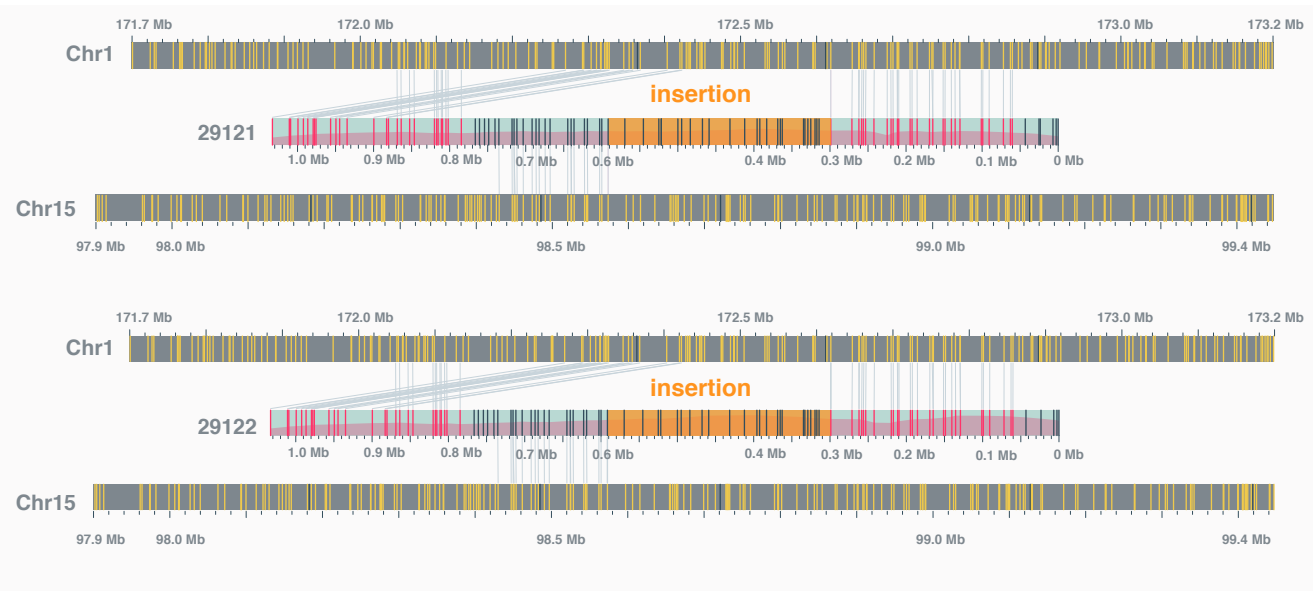

B

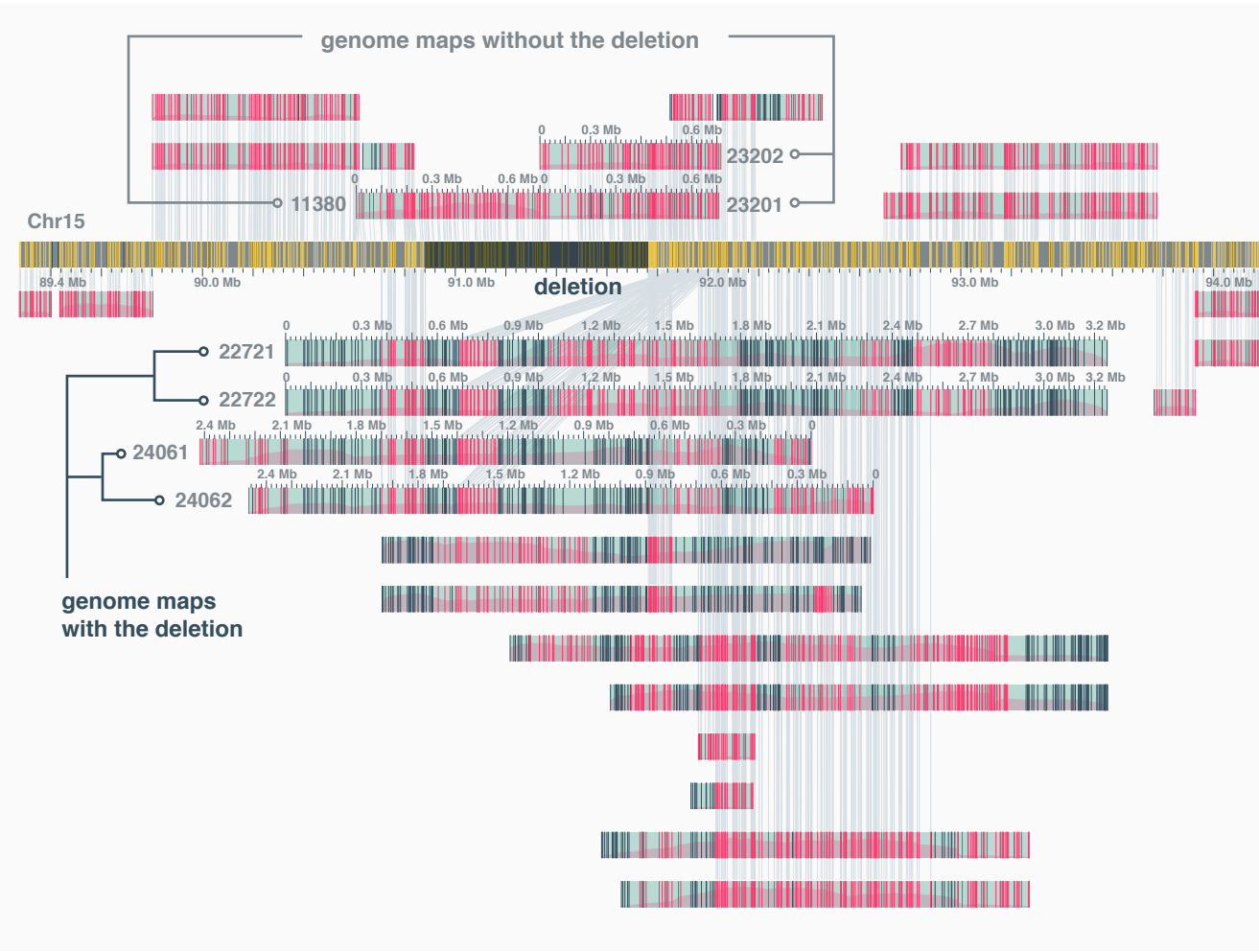

Supplement: Supplemental Material [file supp_gr.227975.117_Supplemental_Fig_S2.ps]

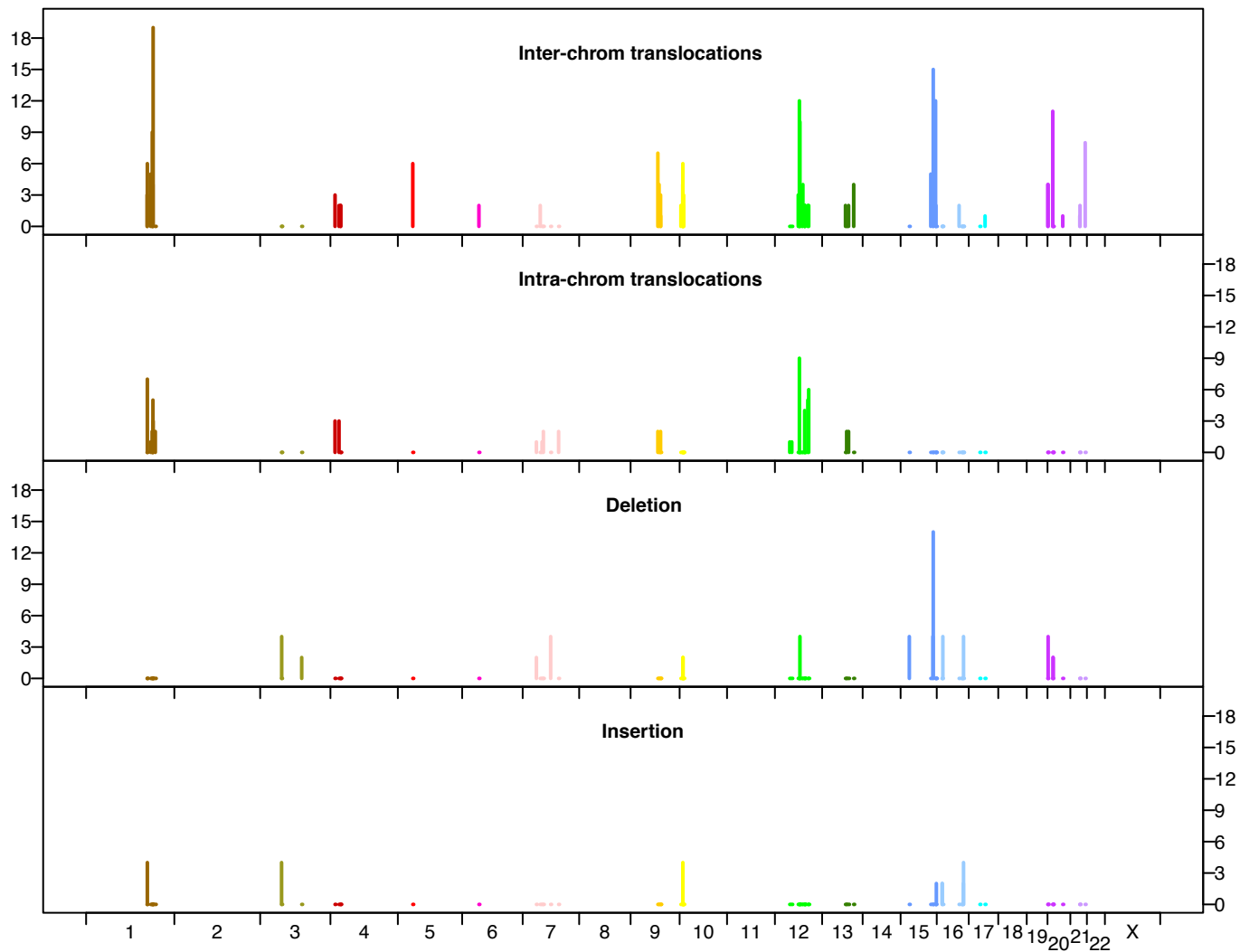

Supplement: Supplemental Material [file supp_gr.227975.117_Supplemental_Fig_S6.ps]

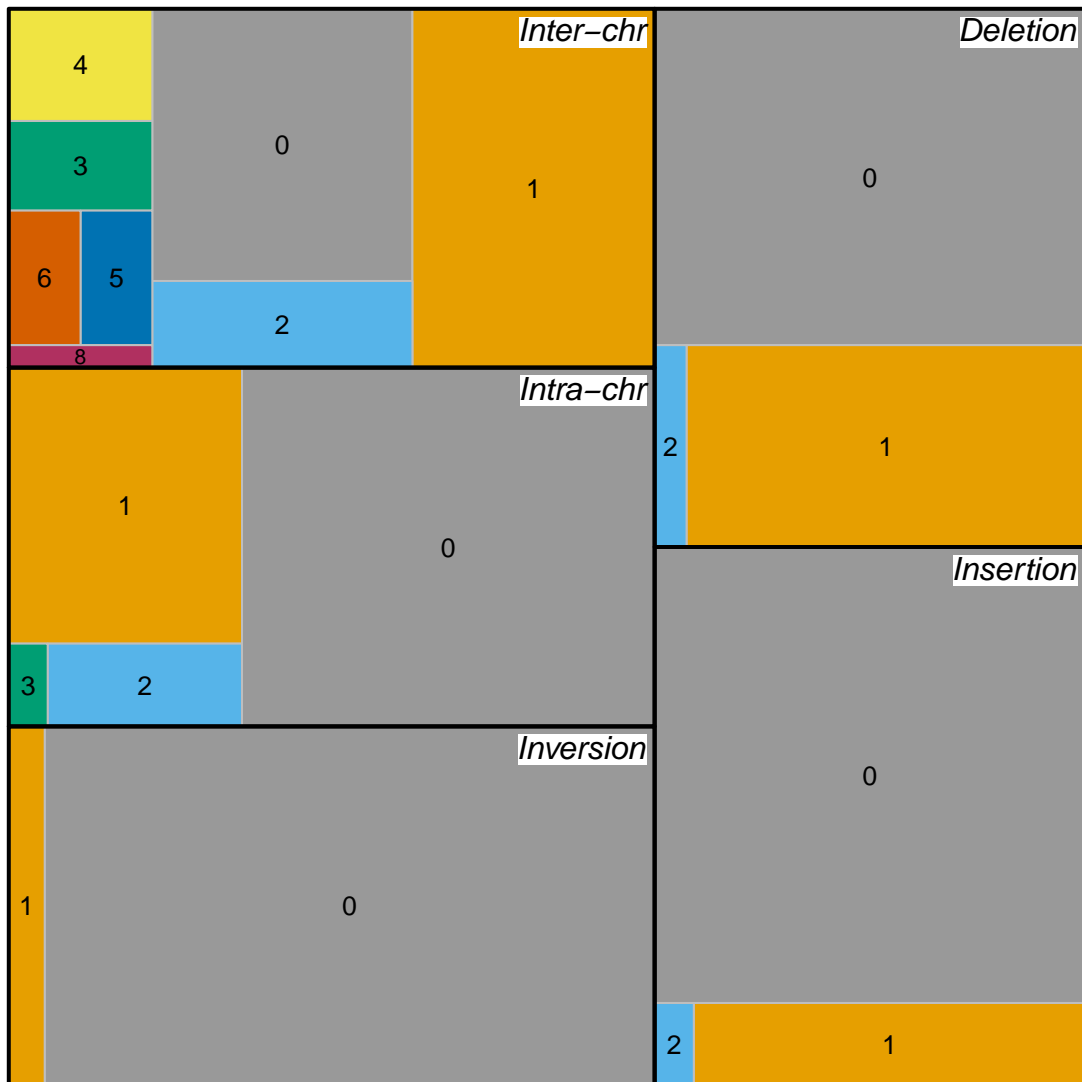

Supplement: Supplemental Material [file supp_gr.227975.117_Supplemental_Fig_S4.pdf]
