# Supplementary material for: Optical mapping reveals a higher level of genomic architecture of chained fusions in cancer
Source: Genome Res. 2018 May;28(5):726–38. doi: 10.1101/gr.227975.117 (PMC5932612; doi:10.1101/gr.227975.117)
Supplement: Supplemental Material [file supp_gr.227975.117_Supplemental_Material.pdf]

Optical mapping reveals a higher level of genomic architecture of chained fusions in cancer

Eva K.F. Chan[1,2,*], Daniel L. Cameron[3,4], Desiree C. Petersen[1,2], Ruth J. Lyons[1], Benedetta F. Baldi[1], Anthony T. Papenfuss[3,4,5,6,7], David M. Thomas[8,9], and Vanessa M. Hayes[1,2,10,11,12]

Supplemental Material

Supplemental Material 1
Supplemental Data 2
Data S1. Bionano data files. 2
Supplemental Tables 2
Table S1. Complex genomic rearrangements in liposarcoma cell line 778. 2
Table S2. GRIDSS results. 2
Supplemental Figures 3
Figure S1. Extent of insertions and deletions found in two normal samples. 3
Figure S2. Largest insertion and deletion found in liposarcoma cell line 778. 5
Figure S3. Liposarcoma cell line 778 fusion maps. 6
Figure S4. Number of structural variant types per fusion map. 7
Figure S5. Sizes of insertion and deletion found in the 72 fusion maps of cell line 778. 8
Figure S6. Genomic distribution of structural variations found in 72 fusion maps of cell line 778. 9
Figure S7. Genome map label patterns of inter-chromosomal translocation junctions and inserted fragments. 11

Supplemental Data

Data S1. Bionano data files. This is a compressed archive containing (i) the input data to Bionano Solve v3.0 (file: all.bnx); (ii) parameter file used (file: exp_optArguments.xml), (iii) the output map file from the *de novo* assembly (file: EXP_REFINEFINAL1.cmap), (iv) alignment files of the consensus genome map set to GRCh38 (files: EXP_REFINEFINAL1.xmap, EXP_REFINEFINAL1_q.cmap, EXP_REFINEFINAL1_r.cmap), and (v) the Variant Annotation Pipeline annotated structural variant file (file: variants_combine_filters_inMoleRefine1.smap). File format specifications are detailed on the Bionano website: https://bionanogenomics.com/support-page/bionano-solve. This data can be access with the following Digital Object Identifier (doi): http://dx.doi.org/10.6070/H41R6P37

Supplemental Tables

Table S1. Complex genomic rearrangements in liposarcoma cell line 778. This is a Microsoft Excel file with three spreadsheets. The spreadsheet named "Garsed Fusions" is a modification of the original Table S2 published in Garsed *et al*. 2014. Additional information relevant to this study include (i) remapping of fusion breakpoint coordinates from GRCh37 to GRCh38; (ii) indication of whether the fusions and their breakpoints are within segmental duplications; (ii) genome map complex genomic rearrangements within 100 kb of the corresponding fusion. The spreadsheet titled "Mapping WG Filtered SV" contains the list of 2,070 filtered structural variant calls from optical mapping of the 778 cell line (it is derived from the variants_combine_filters_inMoleRefine1.smap file of **Supplemental Data S1**; see Methods for details on filtering). "Fusion Map Alignments" lists the 180 breakpoints present in the 72 fusion maps.

Table S2. GRIDSS results. This is a Microsoft Excel file with three spreadsheets. The "optical_mapping_breakpoints" and "gridss_breakpoints" spreadsheets contain candidate fusion breakpoints in BEDPE format. The former contains coordinates of the optical mapping rearrangement coordinates lifted over to GRCh37 (Supplemental Table S1) and the latter contains the QUAL >= 500 breakpoints called by GRIDSS 1.3.4 on the Garsed *et al*. 2014 BAM files. The "matches" spreadsheet lists the GRIDSS breakpoints traversed to verify the corresponding optical mapping fusion as well as the difference in sequence lengths between the GRIDSS breakpoint traversal path and the optical mapping gap size.

Supplemental Figures

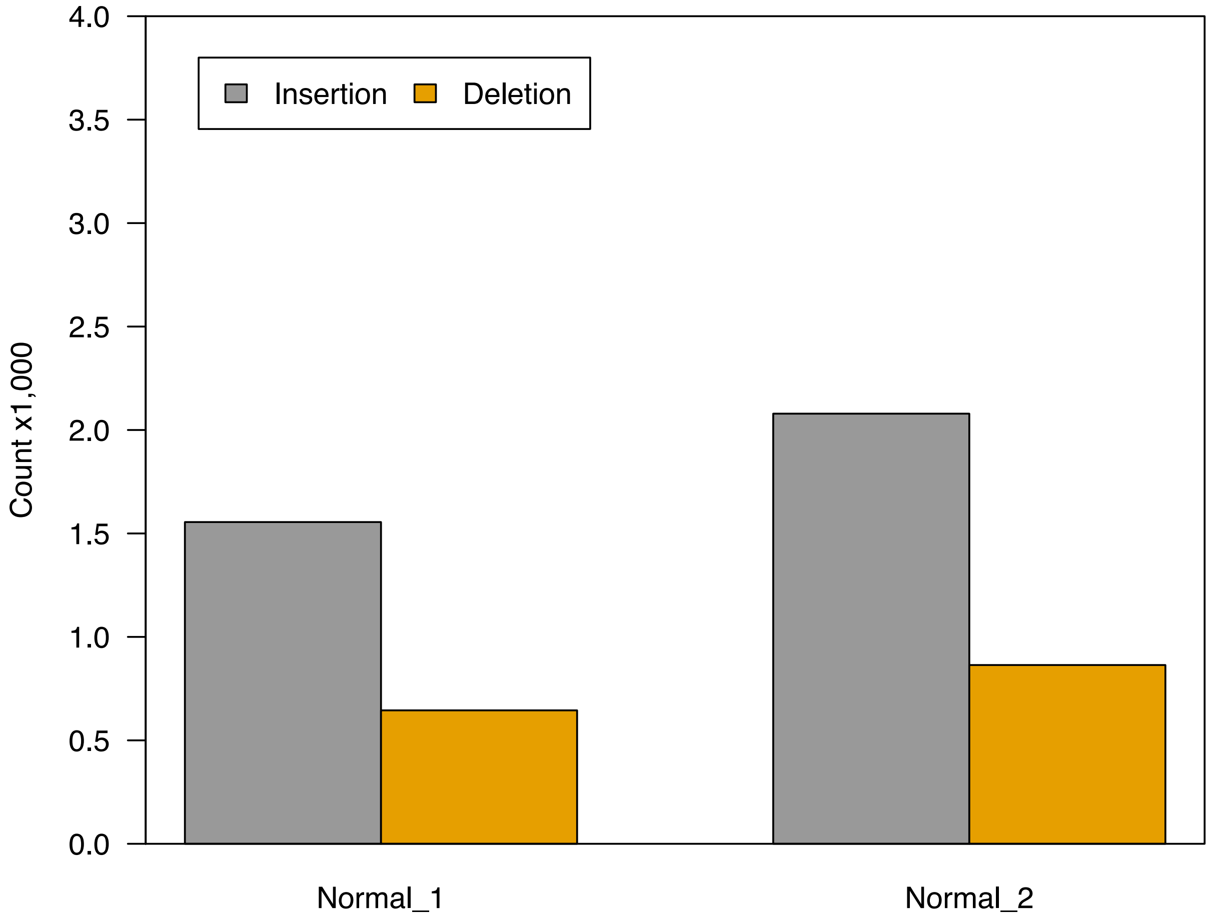

Figure S1. Extent of insertions and deletions found in two normal samples. Shown is a barplot of the number of large (> 1 kb) insertions and deletions found in two whole blood samples from two healthy unrelated individuals.

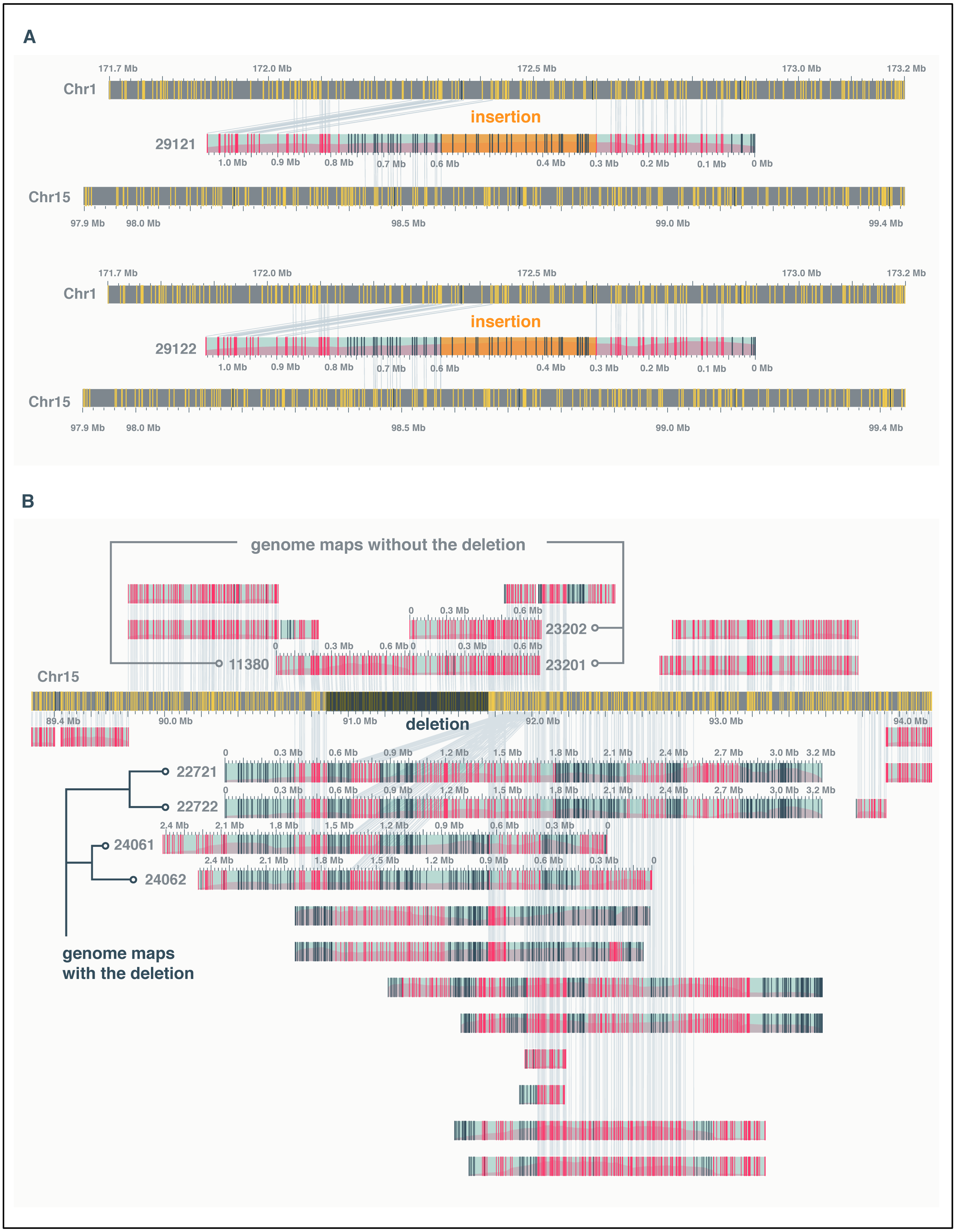
A
Chr1
insertion
29121
Chr15
Chr1
insertion
29122
Chr15
B
genome maps without the deletion
23202
11380
23201
Chr15
deletion
22721
22722
24061
24062
genome maps
with the deletion

Figure S2. Largest insertion and deletion found in liposarcoma cell line 778. (A) The largest insertion was identified in a haplomap pair (#29121/#29122) within Chr1: 172,422 - 172,618 kb. Alignment of the inserted fragment shows a chained fusion of a 84.2 kb fragment from Chromosome 1: 188.314-188.394 Mb, a 143.6 kb fragment from Chromosome 15: 98.430-98.573 Mb) and a 354 kb fragment not aligned to GRCh38. **(B)** The largest deletion of 850 kb at Chr15: 90,881 - 91,870 kb was detected in two haplomap pairs (#22721/#22722 and #24061/#24-62). Three genome maps (#11380 and #23201/#23202) were found to align to the deleted interval, suggesting this Chromosome 15 fragment is not lost to the cell line. Color conventions for this figure is the same as for Fig. 1. Specifically, sample consensus genome maps are represented as teal colored horizontal bars, overlaid with coverage density plots in mauve, while the GRCh38 reference is shown as grey horizontal bars. Fluorescent labels of the Nt.BspQI motif are shown as yellow and pink vertical lines overlaid on the reference and sample genome maps respectively. Labels on the reference not aligned to any consensus genome map and labels on sample genome maps not aligned to the displayed reference chromosome(s) are shown in dark blue, while matching labels are connected by light grey colored lines.

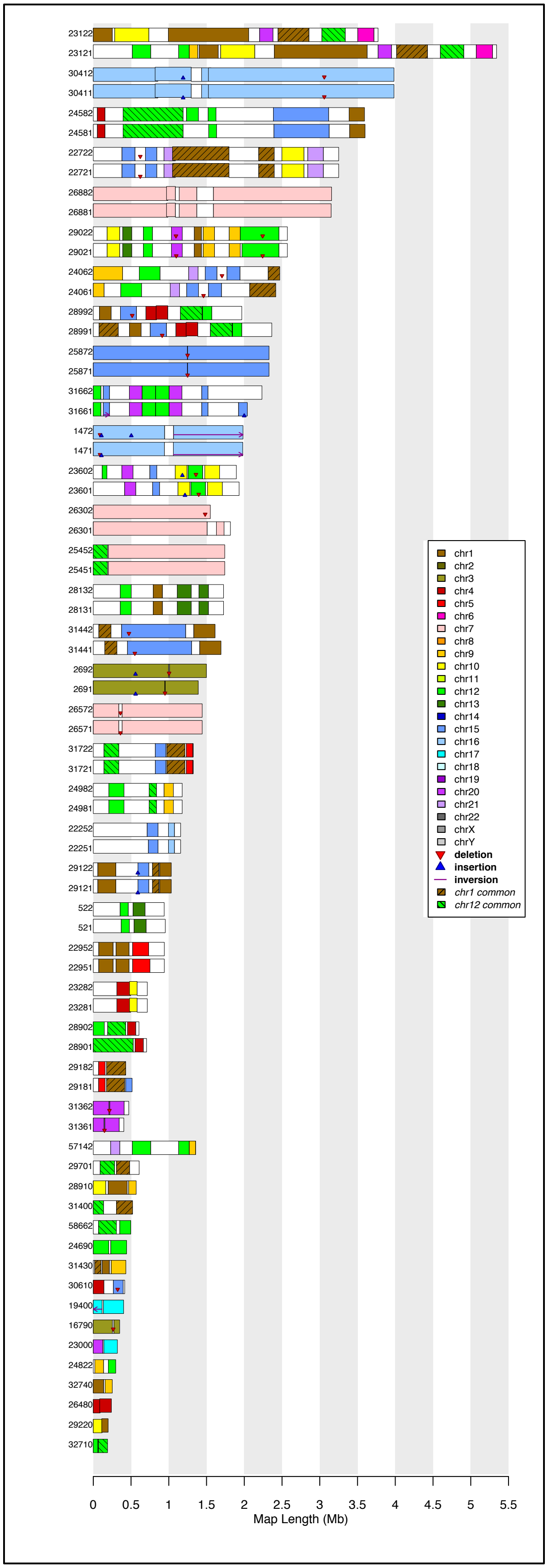

Figure S3. Liposarcoma cell line 778 fusion maps. Shown are 72 consensus genome maps identified in cell line 778 containing complex genomic rearrangements, represented as horizontal rectangles. For each genome map, with map ID shown to the left, regions aligned to the GRCh38 reference are colored per the default UCSC color scheme. Regions not aligned to GRCh38 are not colored (white). Translocations are characterized by alignment junctions, while insertions, deletions, and inversions are indicated by upward blue triangles, downward red triangles, and purple arrows, respectively. Two reference regions (Chr12:68,713,897-69,940,974 and Chr1:188,188,529-189,139,998) found in >40% of the fusion maps, and appear to be translocation hotspots, are highlighted with diagonal stripes. Genome maps are drawn to scale (x-axis) and ordered first by whether they have a haplomap pair, than by their length. Three examples from this figure are highlighted in **Figure 4** in the main text.

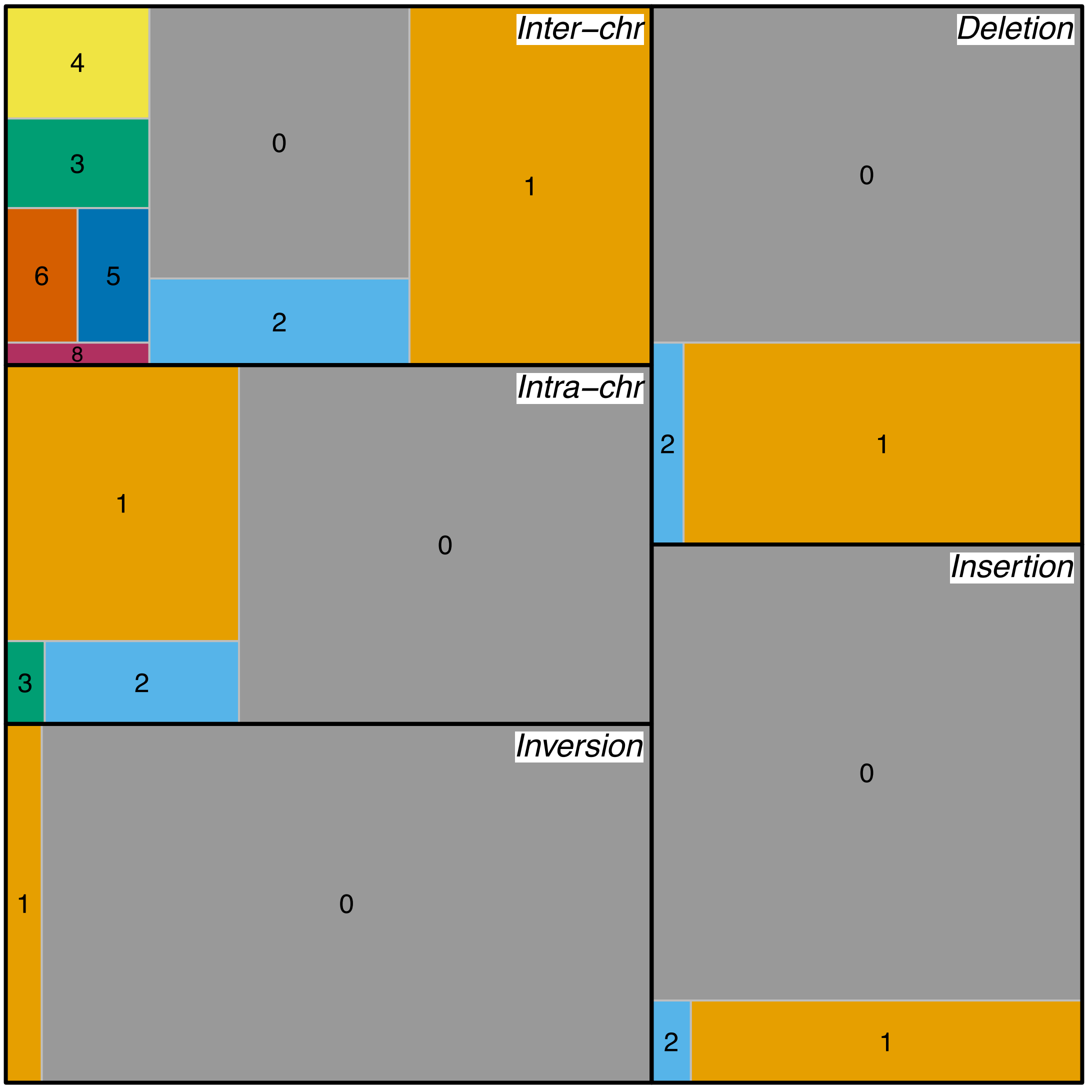

Figure S4. Number of structural variant types per fusion map. Shown is a treemap of the fraction of 72 fusion maps harboring zero to eight of each of the five structural variation types: inter-chromosomal translocation (inter-chr), intra-chromosomal translocation (intra-chr), inversion, deletion, and insertion.

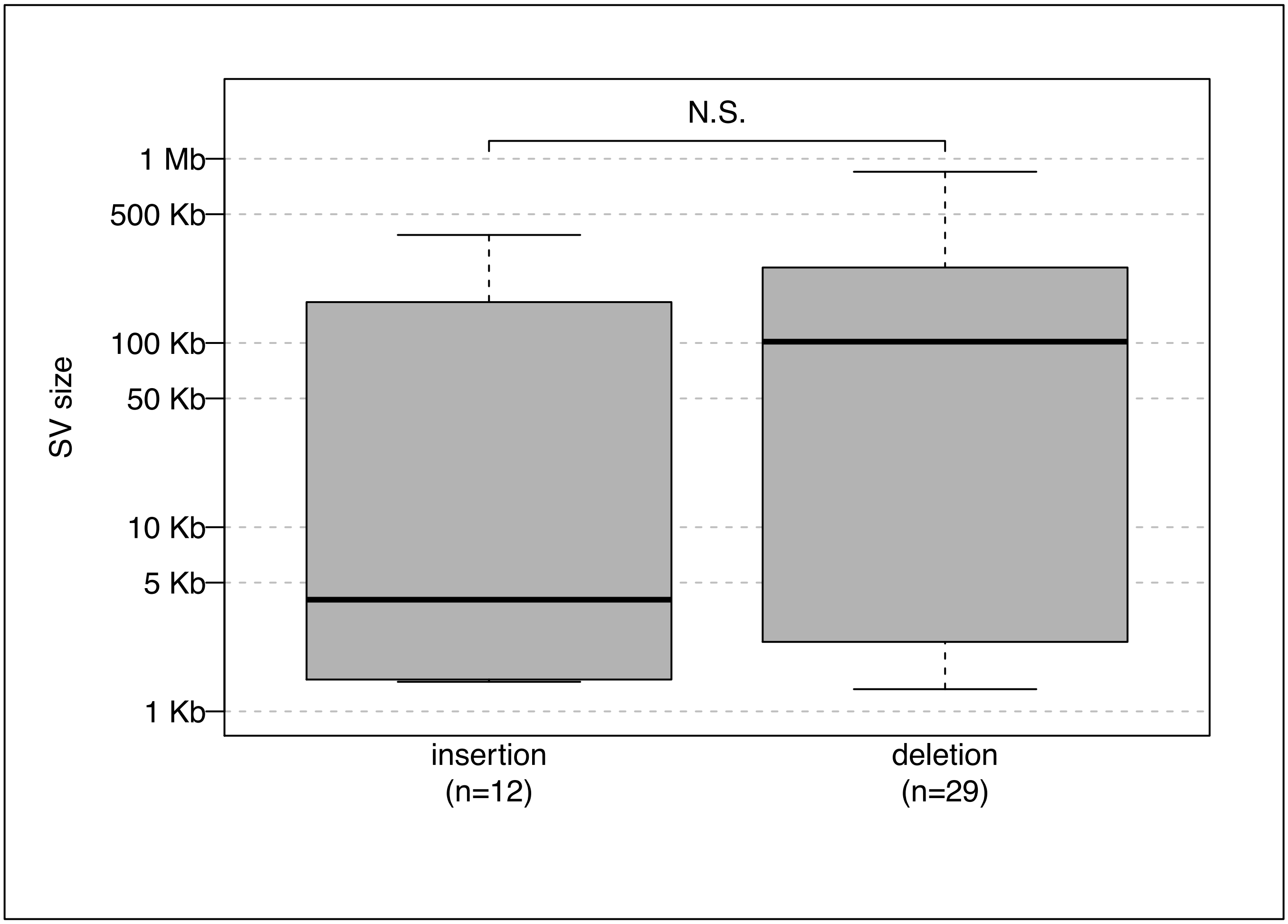

Figure S5. Sizes of insertion and deletion found in the 72 fusion maps of cell line 778. Boxplot showing no significant difference between deletion and insertion sizes, for structural variants larger than 1 kb present in the 72 fusion maps. Thick black horizontal lines in the middle of the boxplots correspond to median values, while shaded gray boxes encompass the interquartile ranges. Structural variant sizes are calculated as the absolute difference in alignment intervals between the reference (GRCh38) and sample (cell line 778) genome maps.

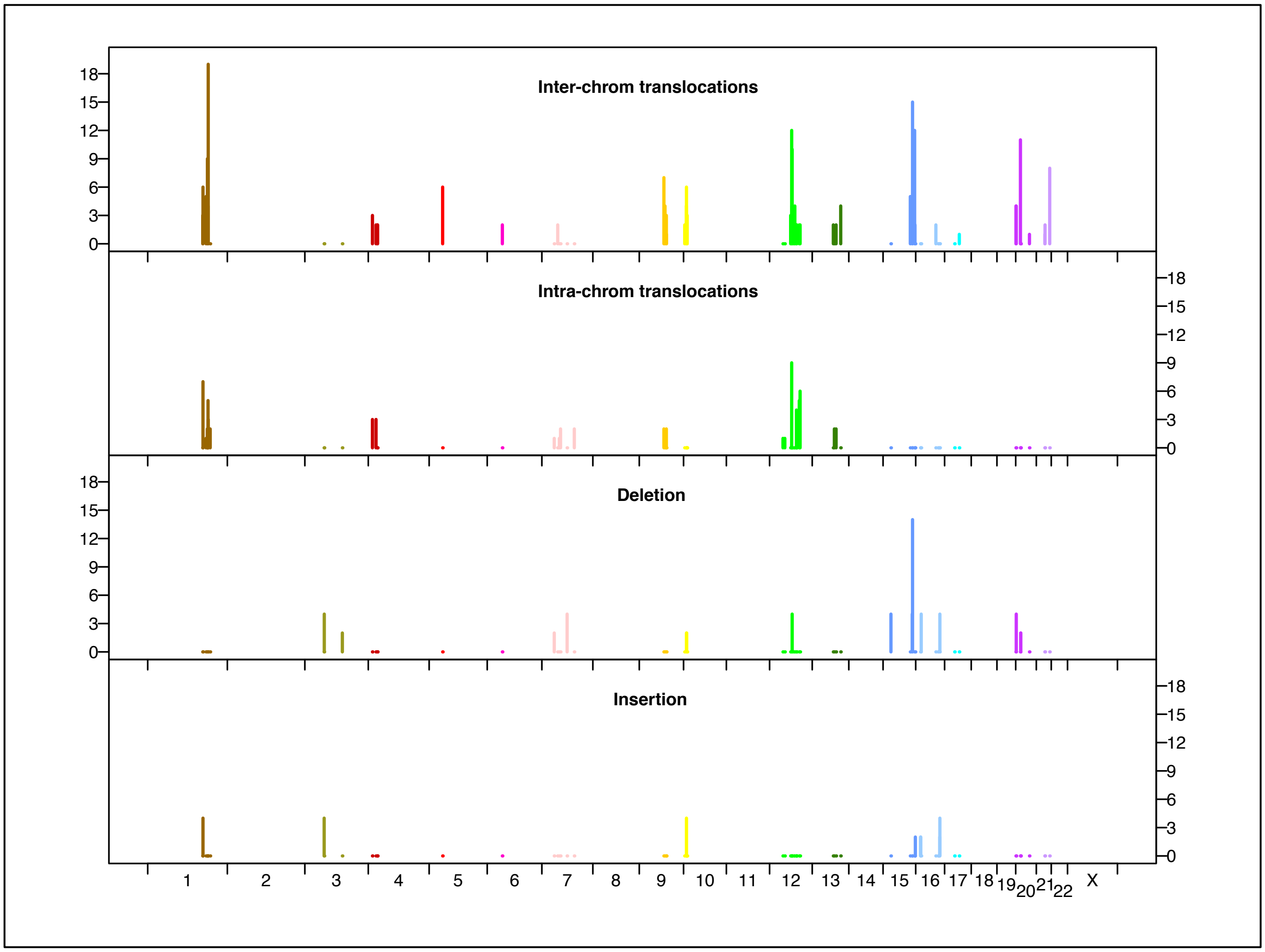

Figure S6. Genomic distribution of structural variations found in 72 fusion maps of cell line 778. Shown are the total numbers of inter-chromosomal translations, intra-chromosomal translocations, deletions, and insertions, per 1 Mb bins along the GRCh38 reference genome. Histogram colors correspond to the default UCSC color schema for human chromosomes.

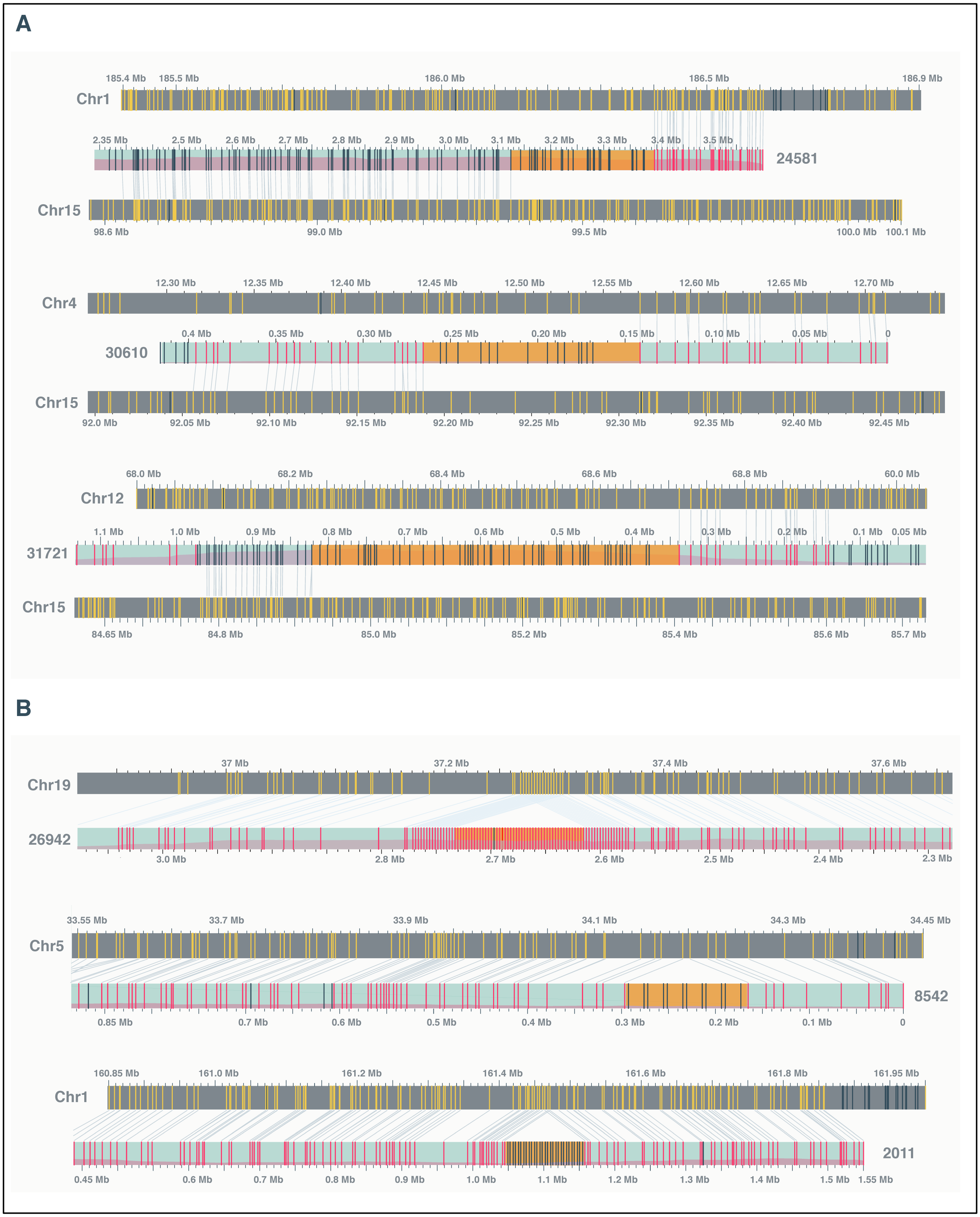
A
185.4 Mb 185.5 Mb 186.0 Mb 186.5 Mb 186.9 Mb
Chr1
2.35 Mb 2.5 Mb 2.6 Mb 2.7 Mb 2.8 Mb 2.9 Mb 3.0 Mb 3.1 Mb 3.2 Mb 3.3 Mb 3.4 Mb 3.5 Mb
24581
Chr15
98.6 Mb 99.0 Mb 99.5 Mb 100.0 Mb 100.1 Mb
12.30 Mb 12.35 Mb 12.40 Mb 12.45 Mb 12.50 Mb 12.55 Mb 12.60 Mb 12.65 Mb 12.70 Mb
Chr4
0.4 Mb 0.35 Mb 0.30 Mb 0.25 Mb 0.20 Mb 0.15 Mb 0.10 Mb 0.05 Mb 0
30610
Chr15
92.0 Mb 92.05 Mb 92.10 Mb 92.15 Mb 92.20 Mb 92.25 Mb 92.30 Mb 92.35 Mb 92.40 Mb 92.45 Mb
68.0 Mb 68.2 Mb 68.4 Mb 68.6 Mb 68.8 Mb 60.0 Mb
Chr12
1.1 Mb 1.0 Mb 0.9 Mb 0.8 Mb 0.7 Mb 0.6 Mb 0.5 Mb 0.4 Mb 0.3 Mb 0.2 Mb 0.1 Mb 0.05 Mb
31721
Chr15
84.65 Mb 84.8 Mb 85.0 Mb 85.2 Mb 85.4 Mb 85.6 Mb 85.7 Mb
B
37 Mb 37.2 Mb 37.4 Mb 37.6 Mb
Chr19
26942
3.0 Mb 2.8 Mb 2.7 Mb 2.6 Mb 2.5 Mb 2.4 Mb 2.3 Mb
33.55 Mb 33.7 Mb 33.9 Mb 34.1 Mb 34.3 Mb 34.45 Mb
Chr5
8542
0.85 Mb 0.7 Mb 0.6 Mb 0.5 Mb 0.4 Mb 0.3 Mb 0.2 Mb 0.1 Mb 0
160.85 Mb 161.0 Mb 161.2 Mb 161.4 Mb 161.6 Mb 161.8 Mb 161.95 Mb
Chr1
2011
0.45 Mb 0.6 Mb 0.7 Mb 0.8 Mb 0.9 Mb 1.0 Mb 1.1 Mb 1.2 Mb 1.3 Mb 1.4 Mb 1.5 Mb 1.55 Mb

Figure S7. Genome map label patterns of inter-chromosomal translocation junctions and inserted fragments. From this study, we observed that, label patterns at the rearrangement junctions are generally "complex" **(A)**, whereas label patterns of inserted fragments tend to be repetitive **(B)**. Insertions of repetitive fragments tend to corresponding with known segmental duplications and are commonly observed control (non disease) samples. Three examples are show for each case. Color schema is per convention detailed in Supplemental Figure S2. Unaligned regions on a genome map are highlighted in orange. An example from each panel has been reproduced in **Figure 6** in the main text.
